# The *Sorcerer II* Global Ocean Sampling Expedition: Northwest Atlantic through Eastern Tropical Pacific

**DOI:** 10.1371/journal.pbio.0050077

**Published:** 2007-03-13

**Authors:** Douglas B Rusch, Aaron L Halpern, Granger Sutton, Karla B Heidelberg, Shannon Williamson, Shibu Yooseph, Dongying Wu, Jonathan A Eisen, Jeff M Hoffman, Karin Remington, Karen Beeson, Bao Tran, Hamilton Smith, Holly Baden-Tillson, Clare Stewart, Joyce Thorpe, Jason Freeman, Cynthia Andrews-Pfannkoch, Joseph E Venter, Kelvin Li, Saul Kravitz, John F Heidelberg, Terry Utterback, Yu-Hui Rogers, Luisa I Falcón, Valeria Souza, Germán Bonilla-Rosso, Luis E Eguiarte, David M Karl, Shubha Sathyendranath, Trevor Platt, Eldredge Bermingham, Victor Gallardo, Giselle Tamayo-Castillo, Michael R Ferrari, Robert L Strausberg, Kenneth Nealson, Robert Friedman, Marvin Frazier, J. Craig Venter

**Affiliations:** 1 J. Craig Venter Institute, Rockville, Maryland, United States of America; 2 Department of Biological Sciences, University of Southern California, Avalon, California, United States of America; 3 Genome Center, University of California Davis, Davis, California, United States of America; 4 Your Genome, Your World, Rockville, Maryland, United States of America; 5 Departmento de Ecología Evolutiva, Instituto de Ecología, Universidad Nacional Autónoma de México, Mexico City, Mexico; 6 Department of Oceanography, University of Hawaii, Honolulu, Hawaii, United States of America; 7 Bedford Institute of Oceanography, Dartmouth, Nova Scotia, Canada; 8 Smithsonian Tropical Research Institute, Balboa, Ancon, Republic of Panama; 9 Departamento de Oceanografía, Universidad de Concepción, Concepción, Chile; 10 Escuela de Química, Universidad de Costa Rica, San Pedro, Costa Rica; 11 Department of Environmental Sciences, Rutgers University, New Brunswick, New Jersey, United States of America; 12 Department of Earth Sciences, University of Southern California, Los Angles, California, United States of America; University of Arizona, United States of America

## Abstract

The world's oceans contain a complex mixture of micro-organisms that are for the most part, uncharacterized both genetically and biochemically. We report here a metagenomic study of the marine planktonic microbiota in which surface (mostly marine) water samples were analyzed as part of the *Sorcerer II* Global Ocean Sampling expedition. These samples, collected across a several-thousand km transect from the North Atlantic through the Panama Canal and ending in the South Pacific yielded an extensive dataset consisting of 7.7 million sequencing reads (6.3 billion bp). Though a few major microbial clades dominate the planktonic marine niche, the dataset contains great diversity with 85% of the assembled sequence and 57% of the unassembled data being unique at a 98% sequence identity cutoff. Using the metadata associated with each sample and sequencing library, we developed new comparative genomic and assembly methods. One comparative genomic method, termed “fragment recruitment,” addressed questions of genome structure, evolution, and taxonomic or phylogenetic diversity, as well as the biochemical diversity of genes and gene families. A second method, termed “extreme assembly,” made possible the assembly and reconstruction of large segments of abundant but clearly nonclonal organisms. Within all abundant populations analyzed, we found extensive intra-ribotype diversity in several forms: (1) extensive sequence variation within orthologous regions throughout a given genome; despite coverage of individual ribotypes approaching 500-fold, most individual sequencing reads are unique; (2) numerous changes in gene content some with direct adaptive implications; and (3) hypervariable genomic islands that are too variable to assemble. The intra-ribotype diversity is organized into genetically isolated populations that have overlapping but independent distributions, implying distinct environmental preference. We present novel methods for measuring the genomic similarity between metagenomic samples and show how they may be grouped into several community types. Specific functional adaptations can be identified both within individual ribotypes and across the entire community, including proteorhodopsin spectral tuning and the presence or absence of the phosphate-binding gene *PstS*.

## Author Summary

Marine microbes remain elusive and mysterious, even though they are the most abundant life form in the ocean, form the base of the marine food web, and drive energy and nutrient cycling. We know so little about the vast majority of microbes because only a small percentage can be cultivated and studied in the lab. Here we report on the Global Ocean Sampling expedition, an environmental metagenomics project that aims to shed light on the role of marine microbes by sequencing their DNA without first needing to isolate individual organisms. A total of 41 different samples were taken from a wide variety of aquatic habitats collected over 8,000 km. The resulting 7.7 million sequencing reads provide an unprecedented look at the incredible diversity and heterogeneity in naturally occurring microbial populations. We have developed new bioinformatic methods to reconstitute large portions of both cultured and uncultured microbial genomes. Organism diversity is analyzed in relation to sampling locations and environmental pressures. Taken together, these data and analyses serve as a foundation for greatly expanding our understanding of individual microbial lineages and their evolution, the nature of marine microbial communities, and how they are impacted by and impact our world.

## Abstract 3

The*Sorcerer II* GOS expedition, data sampling, and analysis is described. The immense diversity in the sequence data required novel comparative genomic assembly methods, which uncovered genomic differences that marker-based methods could not.

## Introduction

**Figure:**
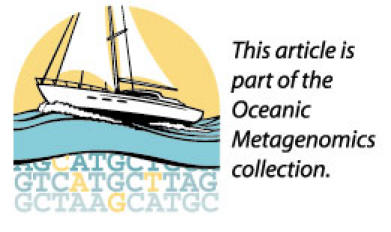


The concept of microbial diversity is not well defined. It can either refer to the genetic (taxonomic or phylogenetic) diversity as commonly measured by molecular genetics methods, or to the biochemical (physiological) diversity measured in the laboratory with pure or mixed cultures. However, we know surprisingly little about either the genetic or biochemical diversity of the microbial world [1], in part because so few microbes have been grown under laboratory conditions [2,3], and also because it is likely that there are immense numbers of low abundance ribotypes that have not been detected using molecular methods [4]. Our understanding of microbial physiological and biochemical diversity has come from studying the less than 1% of organisms that can be maintained in enrichments or cultivated, while our understanding of phylogenetic diversity has come from the application of molecular techniques that are limited in terms of identifying low-abundance members of the communities.

Historically, there was little distinction between genetic and biochemical diversity because our understanding of genetic diversity was based on the study of cultivated microbes. Biochemical diversity, along with a few morphological features, was used to establish genetic diversity via an approach called numerical taxonomy [5,6]. In recent years the situation has dramatically changed. The determination of genetic diversity has relied almost entirely on the use of gene amplification via PCR to conduct taxonomic environmental gene surveys. This approach requires the presence of slowly evolving, highly conserved genes that are found in otherwise very diverse organisms. For example, the gene encoding the small ribosomal subunit RNA, known as 16S, based on sedimentation coefficient, is most often used for distinguishing bacterial and archaeal species [7–10]. The 16S rRNA sequences are highly conserved and can be used as a phylogenetic marker to classify organisms and place them in evolutionary context. Organisms whose 16S sequences are at least 97% identical are commonly considered to be the same ribotype [11], otherwise referred to as species, operational taxonomic units, or phylotypes.

Although rRNA-based analysis has revolutionized our view of genetic diversity, and has allowed the analysis of a large part of the uncultivated majority, it has been less useful in predicting biochemical diversity. Furthermore, the relationship between genetic and biochemical diversity, even for cultivated microbes, is not always predictable or clear. For instance, organisms that have very similar ribotypes (97% or greater homology) may have vast differences in physiology, biochemistry, and genome content. For example, the gene complement of Escherichia coli O157:H7 was found to be substantially different from the K12 strain of the same species [12].

In this paper, we report the results of the first phase of the *Sorcerer II* Global Ocean Sampling (GOS) expedition, a metagenomic study designed to address questions related to genetic and biochemical microbial diversity. This survey was inspired by the British Challenger expedition that took place from 1872–1876, in which the diversity of macroscopic marine life was documented from dredged bottom samples approximately every 200 miles on a circumnavigation [13–15]. Through the substantial dataset described here, we identified 60 highly abundant ribotypes associated with the open ocean and aquatic samples. Despite this relative lack of diversity in ribotype content, we confirm and expand upon previous observations that there is tremendous within-ribotype diversity in marine microbial populations [4,7,8,16,17]. New techniques and tools were developed to make use of the sampling and sequencing metadata. These tools include: (1) the fragment recruitment tool for performing and visualizing comparative genomic analyses when a reference sequence is available; (2) new assembly techniques that use metadata to produce assemblies for uncultivated abundant microbial taxa; and (3) a whole metagenome comparison tool to compare entire samples at arbitrary degrees of genetic divergence. Although there is tremendous diversity within cultivated and uncultivated microbes alike, this diversity is organized into phylogenetically distinct groups we refer to as subtypes.

Subtypes can occupy similar environments yet remain genetically isolated from each other, suggesting that they are adapted for different environmental conditions or roles within the community. The variation between and within subtypes consists primarily of nucleotide polymorphisms but includes numerous small insertions, deletions, and hypervariable segments. Examination of the GOS data in these terms sheds light on patterns of evolution and also suggests approaches towards improving the assembly of complex metagenomic datasets. At least some of this variation can be associated with functional characters that are a direct response to the environment. More than 6.1 million proteins, including thousands of new protein families, have been annotated from this dataset (described in the accompanying paper [18]). In combination, these papers bring us closer to reconciling the genetic and biochemical disconnect and to understanding the marine microbial community.

We describe a metagenomic dataset generated from the *Sorcerer II* expedition. The GOS dataset, which includes and extends our previously published Sargasso Sea dataset [19], now encompasses a total of 41 aquatic, largely marine locations, constituting the largest metagenomic dataset yet produced with a total of ~7.7 million sequencing reads. In the pilot Sargasso Sea study, 200 l surface seawater was filtered to isolate microorganisms for metagenomic analysis. DNA was isolated from the collected organisms, and genome shotgun sequencing methods were used to identify more than 1.2 million new genes, providing evidence for substantial microbial taxonomic diversity [19]. Several hundred new and diverse examples of the proteorhodopsin family of light-harvesting genes were identified, documenting their extensive abundance and pointing to a possible important role in energy metabolism under low-nutrient conditions. However, substantial sequence diversity resulted in only limited genome assembly. These results generated many additional questions: would the same organisms exist everywhere in the ocean, leading to improved assembly as sequence coverage increased; what was the global extent of gene and gene family diversity, and can we begin to exhaust it with a large but achievable amount of sequencing; how do regions of the ocean differ from one another; and how are different environmental pressures reflected in organisms and communities? In this paper we attempt to address these issues.

## Results

### Sampling and the Metagenomic Dataset

**Figure 1:**
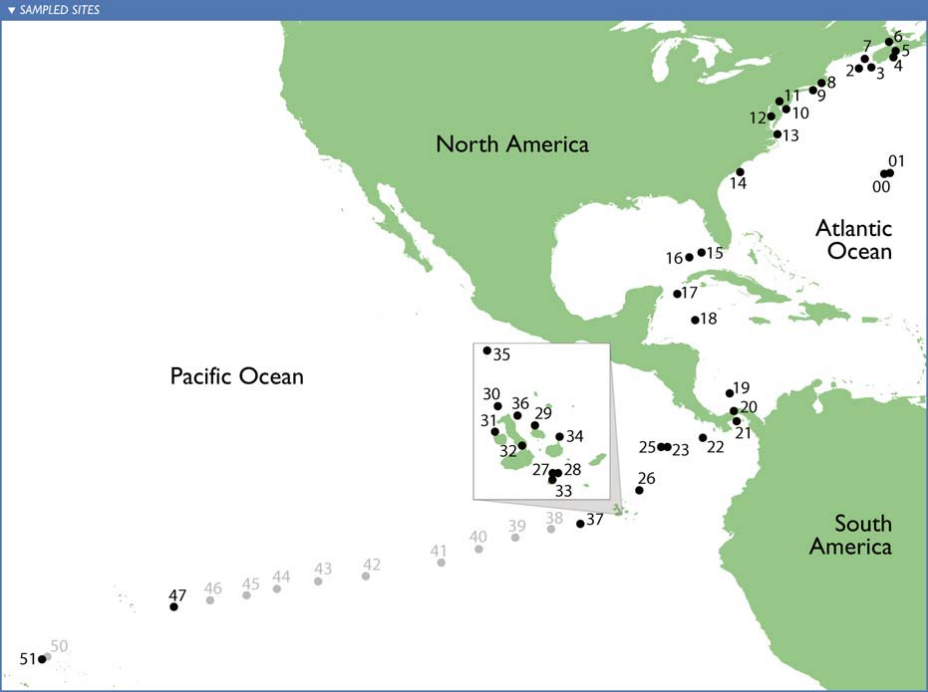
Sampling Sites Microbial populations were sampled from locations in the order shown. Samples were collected at approximately 200 miles (320 km) intervals along the eastern North American coast through the Gulf of Mexico into the equatorial Pacific. Samples 00 and 01 identify sets of sites sampled as part of the Sargasso Sea pilot study [19]. Samples 27 through 36 were sampled off the Galapagos Islands (see inset). Sites shown in gray were not analyzed as part of this study.

Microbial samples were collected as part of the *Sorcerer II* expedition between August 8, 2003, and May 22, 2004, by the S/V *Sorcerer II,* a 32-m sailing sloop modified for marine research. Most specimens were collected from surface water marine environments at approximately 320-km (200-mile) intervals. In all, 44 samples were obtained from 41 sites (Figure 1), covering a wide range of distinct surface marine environments as well as a few nonmarine aquatic samples for contrast (Table 1).

**Table 1:**
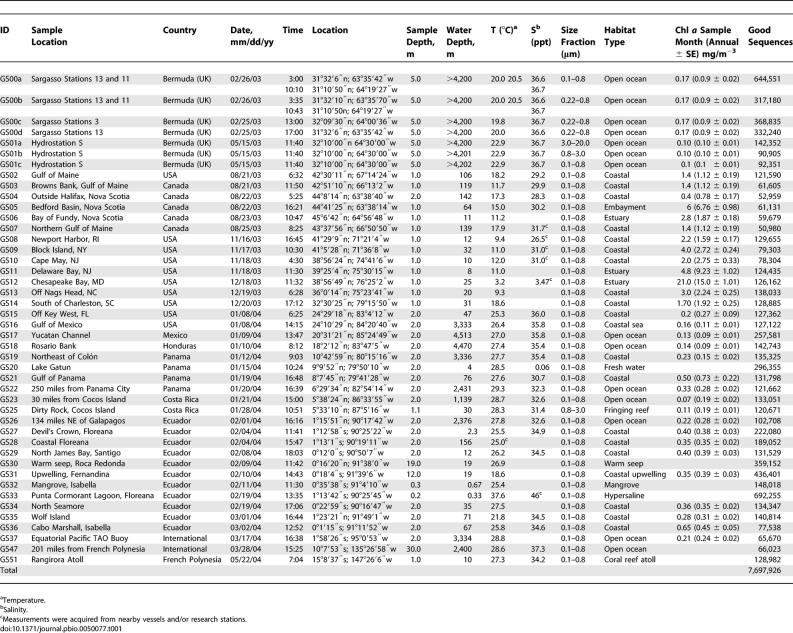
Sampling Locations and Environmental Data

Several size fractions were isolated for every site (see Materials and Methods). Total DNA was extracted from one or more fractions, mostly from the 0.1–0.8-μm size range. This fraction is dominated by bacteria, whose compact genomes are particularly suitable for shotgun sequencing. Random-insert clone libraries were constructed. Depending on the uniqueness of each sampling site and initial estimates of the genetic diversity, between 44,000 and 420,000 clones per sample were end-sequenced to generate mated sequencing reads. In all, the combined dataset includes 6.25 Gbp of sequence data from 41 different locations. Many of the clone libraries were constructed with a small insert size (<2 kbp) to maximize cloning efficiency. As this often resulted in mated sequencing reads that overlapped one another, overlapping mated reads were combined, yielding a total of ~6.4 M contiguous sequences, totaling ~5.9 Gbp of nonredundant sequence. Taken together, this is the largest collection of metagenomic sequences to date, providing more than a 5-fold increase over the dataset produced from the Sargasso Sea pilot study [19] and more than a 90-fold increase over the other large marine metagenomic dataset [20].

### Assembly

Assembling genomic data into larger contigs and scaffolds, especially metagenomic data, can be extremely valuable, as it places individual sequencing reads into a greater genomic context. A largely contiguous sequence links genes into operons, but also permits the investigation of larger biochemical and/or physiological pathways, and also connects otherwise-anonymous sequences with highly studied “taxonomic markers” such as 16S or *recA,* thus clearly identifying the taxonomic group with which they are associated. The primary assembly of the combined GOS dataset was performed using the Celera Assembler [21] with modifications as previously described [19] and as given in Materials and Methods. The assembly was performed with quite stringent criteria, beginning with an overlap cutoff of 98% identity to reduce the potential for artifacts (e.g., chimeric assemblies or consensus sequences diverging substantially from the genome of any given cell). This assembly was the substrate for annotation (see the accompanying paper by Yooseph et al. [18]).

The degree of assembly of a metagenomic sample provides an indication of the diversity of the sample. A few substantial assemblies notwithstanding, the primary assembly was strikingly fragmented (Table 2). Only 9% of sequencing reads went into scaffolds longer than 10 kbp. A majority (53%) of the sequencing reads remained unassembled singletons. Scaffolds containing more than 50 kb of consensus sequence totaled 20.7 Mbp; of these, >75% were produced from a single Sargasso Sea sample and correspond to the *Burkholderia* or *Shewanella* assemblies described previously [19]. These results highlight the unusual abundance of these two organisms in a single sample, which significantly affected our expectations regarding the current dataset. Given the large size of the combined dataset and the substantial amount of sequencing performed on individual filters, the overall lack of assembly provides evidence of a high degree of diversity in surface planktonic communities. To put this in context, suppose there were a clonal organism that made up 1% of our data, or ~60 Mbp. Even a genome of 10 Mbp—enormous by bacterial standards—would be covered ~6-fold. Such data might theoretically assemble with an average contig approaching 50 kb [22]. While real assemblies generally fall short of theory for various reasons, *Shewanella* data make up <1% of the total GOS dataset, and yet most of the relevant reads assemble into scaffolds >50 kb. Thus, with few scaffolds of significant length, we could conclude that there are very few clonal organisms present at even 1% in the GOS dataset.

**Table 2:**
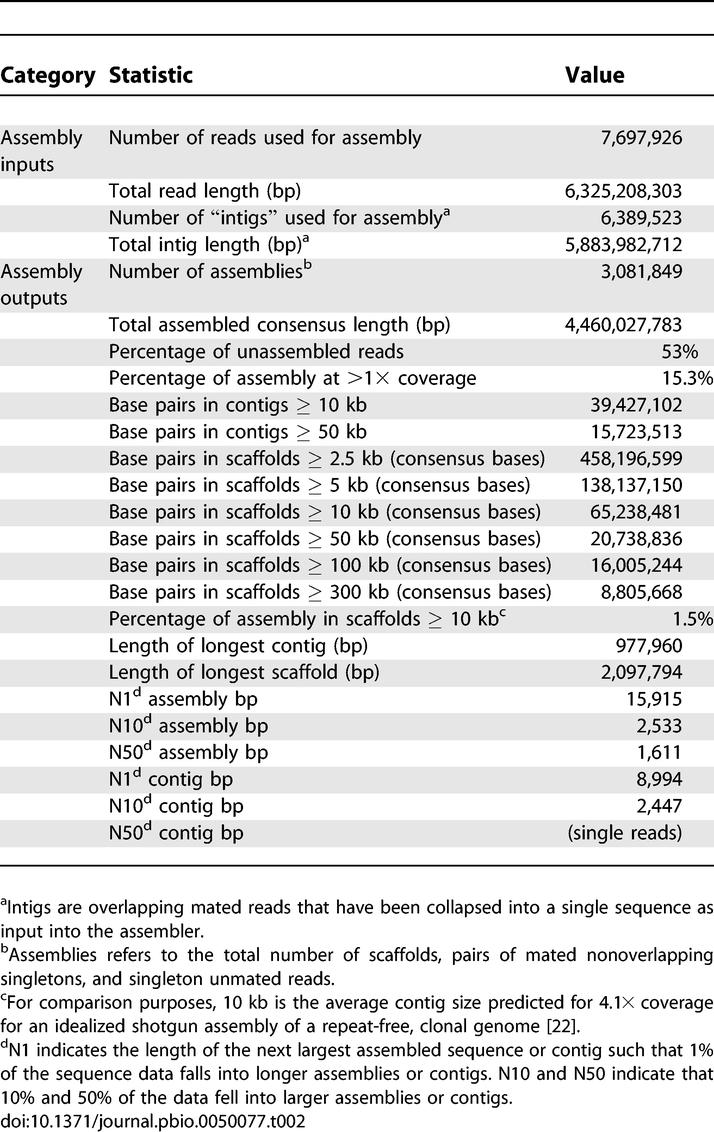
Summary Assembly Statistics

To investigate the nature of the implied diversity and to see whether greater assembly could be achieved, we explored several alternative approaches. Breaks in the primary assembly resulted from two factors: incomplete sequence coverage and conflicts in the data. Conflicts can break assemblies when there is no consistent way to chain together all overlapping sequencing reads. As it was possible that there would be fewer conflicts within a single sample (i.e., that diversity within a single sample would be lower), assemblies were attempted with individual samples. However, the results did not show any systematic improvements even in those samples with greater coverage (unpublished data). Upon manual inspection, most assembly-breaking conflicts were found to be local in nature. These observations suggested that reducing the degree of sequence identity required for assembly could ameliorate both factors limiting assembly: effective coverage would increase and many minor conflicts would be resolved.

Accordingly, we produced a series of assemblies based on 98%, 94%, 90%, 85%, and 80% identity overlaps for two subsets of the GOS dataset, again using the Celera Assembler. Assembly lengths increased as the overlap cutoff decreased from 98% to 94% to 90%, and then leveled off or even dropped as stringency was reduced below 90% (Table 3). Although larger assemblies could be generated using lower identity overlaps, significant numbers of overlaps satisfying the chosen percent identity cutoff still went unused in each assembly. This is consistent with a high rate of conflicting overlaps and in turn diagnostic of significant polymorphism.

**Table 3:**
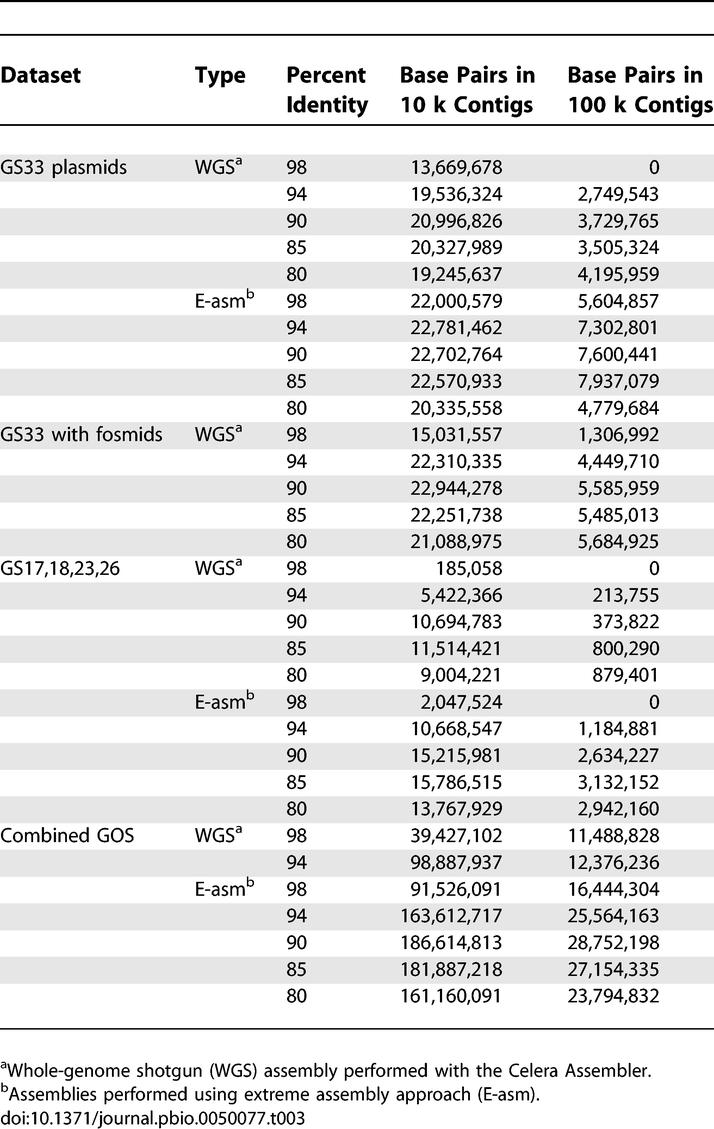
Evaluation of Alternative Assembly Methods

In mammalian sequencing projects the use of larger insert libraries is critical to producing larger assemblies because of their ability to span repeats or local polymorphic regions [23]. The shotgun sequencing libraries from the GOS filters were typically constructed from inserts shorter than 2 kb. Longer plasmid libraries were attempted but were much less stable. We obtained paired-end sequences from 21,419 fosmid clones (average insert size, 36 kb; [24,25]) from the 0.1-micron fraction of GS-33. The effect of these long mate pairs on the GS-33 assembly was quite dramatic, particularly at high stringency (e.g., improving the largest scaffold from 70 kb to 1,247 kb and the largest contig from 70 kb to 427 kb). At least for GS-33 this suggests that many of the polymorphisms affect small, localized regions of the genome that can be spanned using larger inserts. This degree of improvement may be greater than what could be expected in general, as the diversity of GS-33 is by far the lowest of any of the currently sequenced GOS samples, yet it clearly indicates the utility of including larger insert libraries for assembly.

### Fragment Recruitment

In the absence of substantial assembly, direct comparison of the GOS sequencing data to the genomes of sequenced microbes is an alternative way of providing context, and also allows for exploration of genetic variation and diversity. A large and growing set of microbial genomes are available from the National Center for Biotechnology Information (NCBI; http://www.ncbi.nlm.nih.gov). At the time of this study, we used 334 finished and 250 draft microbial genomes as references for comparison with the GOS sequencing reads. Comparisons were carried out in nucleotide-space using the sequence alignment tool BLAST [26]. BLAST parameters were designed to be extremely lenient so as to detect even distant similarities (as low as 55% identity). A large proportion of the GOS reads, 70% in all, aligned to one or more genomes under these conditions. However, many of the alignments were of low identity and used only a portion of the entire read. Such low-quality hits may reflect distant evolutionary relationships, and therefore less information is gained based on the context of the alignment. More stringent criteria could be imposed requiring that the reads be aligned over nearly their entire length without any large gaps. Using this stringent criterion only about 30% of the reads aligned to any of the 584 reference genomes. We refer to these fully aligned reads as “recruited reads.” Recruited reads are far more likely to be from microbes closely related to the reference sequence (same species) than are partial alignments. Despite the large number of microbial genomes currently available, including a large number of marine microbes, these results indicate that a substantial majority of GOS reads cannot be specifically related to available microbial genomes.

**Figure 2:**
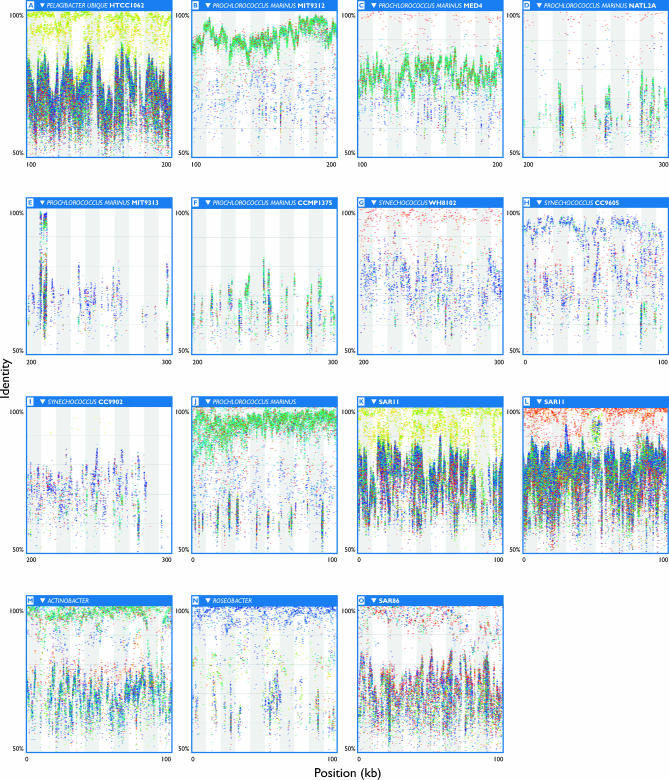
Fragment Recruitment Plots The horizontal axis of each panel corresponds to a 100-kb segment of genomic sequence from the indicated reference microbial genome. The vertical axis indicates the sequence identity of an alignment between a GOS sequence and the reference genomic sequence. The identity ranges from 100% (top) to 50% (bottom). Individual GOS sequencing reads were colored to reflect the sample from which they were isolated. Geographically nearby samples have similar colors (see Poster S1 for key). Each organism shows a distinct pattern of recruitment reflecting its origin and relationship to the environmental data collected during the course of this study. (A) P. ubique HTCC1062 recruits the greatest density of GOS sequences of any genome examined to date. The GOS sequences show geographic stratification into bands, with sequences from temperate water samples off the North American coast having the highest identity (yellow to yellow-green colors). At lower identity, sequences from all the marine environments could be aligned to HTCC1062. (B) P. marinus MIT9312 recruits a large number of GOS sequences into a single band that zigzags between 85%–95% identity on average. These sequences are largely derived from warm water samples in the Gulf of Mexico and eastern Pacific (green to greenish-blue reads). (C) P. marinus MED4 recruits largely the same set of reads as MIT9312 (B) though the sequences that form the zigzag recruit at a substantially lower identity. A small number of sequences from the Sargasso Sea samples (red) are found at high identity. (D) P. marinus NATL2A recruits far fewer sequences than any of the preceding panels. Like MED4, a small number of high-identity sequences were recruited from the Sargasso samples. (E) P. marinus MIT9313 is a deep-water low-light–adapted strain of *Prochlorococcus.* GOS sequences were recruited almost exclusively at low identity in vertical stacks that correspond to the locations of conserved genes. On the left side of this panel is a very distinctive pattern of recruitment that corresponds to the highly conserved 16S and 23S mRNA gene operon. (F) P. marinus CCMP1375, another deep-water low-light–adapted strain, does not recruit GOS sequences at high identity. Only stacks of sequences are seen corresponding to the location of conserved genes. (G) *Synechococcus* WH8102 recruits a modest number of high-identity sequences primarily from the Sargasso Sea samples. A large number of moderate identity matches from the Pacific and hypersaline lagoon (GS33) samples are also visible. (H) *Synechococcus* CC9605 recruits largely the same sequences as does *Synechococcus* WH8102, but was isolated from Pacific waters. GOS sequences from some of the Pacific samples recruit at high identity, while sequences from the Sargasso and hypersaline lagoon (bluish-purple) were recruited at moderate identities. (I) *Synechococcus* CC9902 is distantly related to either of the preceding *Synechococcus* strains. While this strain also recruits largely the same sequences as the WH8102 and CC9902 strains, they recruit at significantly lower identity. (J–O) Fragment recruitment plots to extreme assemblies seeded with phylogenetically informative sequences. Using this approach it is not only possible to assemble contigs with strong similarities to known genomes but to identify contigs from previously uncultured genomes. In each case a 100-kb segment from an extreme assembly is shown. Each plot shows a distinct pattern of recruitment that distinguishes the panels from each other. (J) Seeded from a Prochlorococcus marinus-related sequence, this contig recruits a broad swath of GOS sequences that correspond to the GOS sequences that form the zigzag on P. marinus MIT9312 recruitment plots (see [B] or Poster S1 for comparison). (K–L) Seeded from SAR11 clones, these contigs show significant synteny to the known P. ubique HTCC1062 genome. (K) is strikingly similar to previous recruitment plots to the HTCC1062 genome (see [A] or Poster S1). In contrast, (L) identifies a different strain that recruits high-identity GOS sequences primarily from the Sargasso Sea samples (red). (M–O) These three panels show recruitment plots to contigs belonging to the uncultured *Actinobacter, Roseobacter,* and SAR86 lineages.

The amount and distribution of reads recruited to any given genome provides an indication of the abundance of closely related organisms. Only genomes from the five bacterial genera *Prochlorococcus, Synechococcus, Pelagibacter, Shewanella,* and *Burkholderia* yielded substantial and uniform recruitment of GOS fragments over most of a reference genome (Table 4). These genera include multiple reference genomes, and we observed significant differences in recruitment patterns even between organisms belonging to the same species (Figure 2A–2I). Three genera, *Pelagibacter* (Figure 2A), *Prochlorococcus* (Figure 2B–2F), and *Synechococcus* (Figure 2G–2I), were found abundantly in a wide range of samples and together accounted for roughly 50% of all the recruited reads (though only ~15% of all GOS sequencing reads). By contrast, although every genome tested recruited some GOS reads, most recruited only a small number, and these reads clustered at lower identity to locations corresponding to large highly conserved genes (for typical examples see Figure 2E–2F). We refer to this pattern as nonspecific recruitment as it reflects taxonomically nonspecific signals, with the reads in question often recruiting to distantly related sets of genomes. Most microbial genomes, including many of the marine microbes (e.g., the ubiquitous genus *Vibrio*), demonstrated this nonspecific pattern of recruitment.

**Table 4:**
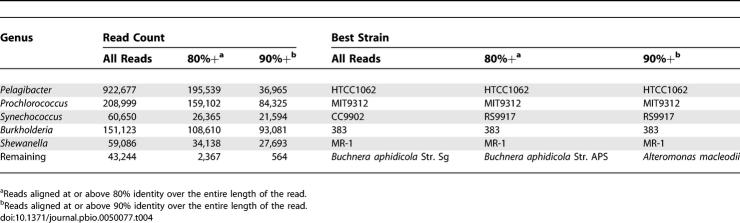
Microbial Genera that Recruited the Bulk of the GOS Reads

The relationship between the similarity of an individual sequencing read to a given genome and the sample from which the read was isolated can provide insight into the structure, evolution, and geographic distribution of microbial populations. These relationships were assessed by constructing a “percent identity plot” [27] in which the alignment of a read to a reference sequence is shown as a bar whose horizontal position indicates location on the reference and whose vertical position indicates the percent identity of the alignment. We colored the plotted reads according to the samples to which they belonged, thus indirectly representing various forms of metadata (geographic, environmental, and laboratory variables). We refer to these plots that incorporate metadata as fragment recruitment plots. Fragment recruitment plots of GOS sequences recruited to the entire genomes of Pelagibacter ubique HTCC1062, Prochlorococcus marinus MIT9312, and *Synechococcus* WH8102 are presented in Poster S1.

### Within-Ribotype Population Structure and Variation

Characteristic patterns of recruitment emerged from each of these abundant marine microbes consisting of horizontal bands made up of large numbers of GOS reads. These bands seem constrained to a relatively narrow range of identities that tile continuously (or at least uniformly, in the case when abundance/coverage is lower) along ~90% of the reference sequence. The uninterrupted tiling indicates that environmental genomes are largely syntenic with the reference genomes. Multiple bands, distinguished by degree of similarity to the reference and by sample makeup, may arise on a single reference (Poster S1D and S1F). Each of these bands appears to represent a distinct, closely related population we refer to as a subtype. In some cases, an abundant subtype is highly similar to the reference genome, as is the case for P. marinus MIT9312 (Poster S1) and *Synechococcus* RS9917 (unpublished data). P. ubique HTCC1062 and other *Synechococcus* strains like WH8102 show more complicated banding patterns (Poster S1D and S1F) because of the presence of multiple subtypes that produce complex often overlapping bands in the plots. Though the recruitment patterns can be quite complex they are also remarkably consistent over much of the reference genome. In these more complicated recruitment plots, such as the one for P. ubique HTCC1062, individual bands can show sudden shifts in identity or disappear altogether, producing a gap in recruitment that appears to be specific to that band (see P. ubique recruitment plots on Poster S1B and S1E, and specifically between 130–140 kb). Finally, phylogenetic analysis indicates that separate bands are indeed evolutionarily distinct at randomly selected locations along the genome.

**Figure 3:**
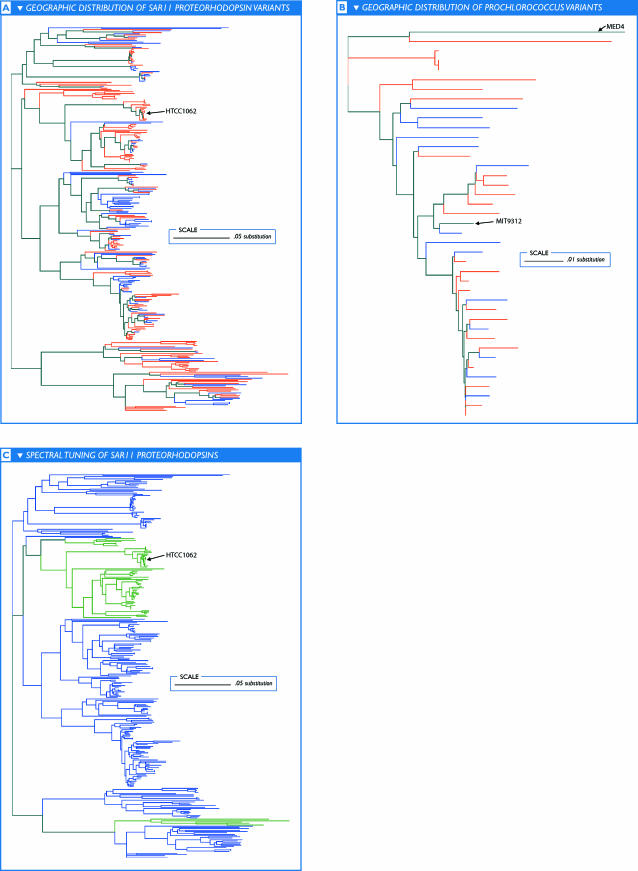
Population Structure and Variation as Revealed by Phylogeny Phylogenies were produced using neighbor-joining. There is significant within-clade variation as well as an absence of strong geographic structure to variants of SAR11 (P. ubique HTCC1062) and P. marinus MIT9312. Similar reads are not necessarily from similar locations, and reads from similar locations are not necessarily similar. (A) Geographic distribution of SAR11 proteorhodopsin variants. Keys to coloration: blue, Pacific; pink, Atlantic. (B) Geographic distribution of *Prochlorococcus* variants. Keys to coloration: blue, Pacific; pink, Atlantic. (C) Origins of spectral tuning of SAR11 proteorhodopsins. Reads are colored according to whether they contain the L (green) or Q (blue) variant at the spectral tuning residue described in the text. The selection of tuning residue is lineage restricted, but each variant must have arisen on two separate occasions.

The amount of sequence variation within a given band cannot be reliably determined from the fragment recruitment plots themselves. To examine this variation, we produced multiple sequence alignments and phylogenies of reads that recruited to several randomly chosen intervals along given reference genomes to show that there can be considerable within-subtype variation (Figure 3A–3B). For example, within the primary band found in recruitment plots to P. marinus MIT9312, individual pairs of overlapping reads typically differ on average between 3%–5% at the nucleotide level (depending on exact location in the genome). Very few reads that recruited to MIT9312 have perfect (mismatch-free) overlaps with any other read or to MIT9312, despite ~100-fold coverage. While many of these differences are silent (i.e., do not change amino acid sequences), there is still considerable variation at the protein level (unpublished data). The amount of variation within subtypes is so great that it is likely that no two sequenced cells contained identical genomes.

### Identifying Genomic Structural Variation with Metagenomic Data

**Figure 4:**
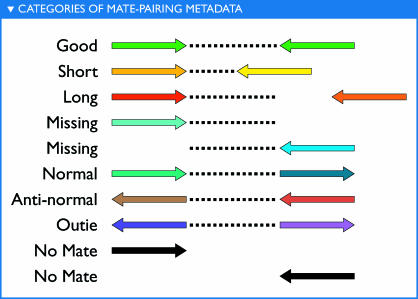
Categories of Recruitment Metadata The recruitment metadata distinguishes eight different general categories based on the relative placement of paired end sequencing reads (mated reads) when recruited to a reference sequence in comparison to their known orientation and separation on the clone from which they were derived. Assuming orientation is correct, two mated reads can be recruited closer together, further apart, or within expected distances given the size of the clone from which the sequences were derived. These sequences are categorized as “short,” “long,” or “good,” respectively. Alternately, the mated reads may be recruited in a mis-oriented fashion, which trumps issues of separation. These reads can be categorized as “normal,” “anti-normal,” or “outie.” In addition, there are two other categories. “No mate” indicates that no mated read was available for recruitment, possibly due to sequencing error. Perhaps most useful of any of the recruitment categories, “missing” mates indicate that while a mated sequence was available, it was not recruited to the reference. “Missing” mates identify breaks in synteny between the environmental data and the reference sequence.

**Figure 5:**
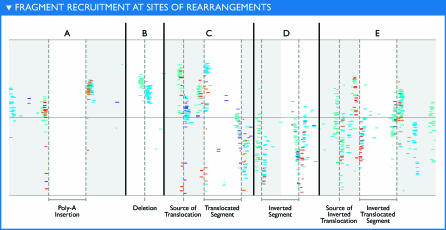
Fragment Recruitment at Sites of Rearrangements Environmental sequences recruited near breaks in synteny have characteristic patterns of recruitment metadata. Indeed, each of five basic rearrangements (i.e., insertion, deletion, translocation, inversion, and inverted translocation) produced a distinct pattern when examining the recruitment metadata. Here, example recruitment plots for each type of rearrangement have been artificially generated. The “good” and “no mate” categories have been suppressed. In each case, breaks in synteny are marked by the presence of stacks of “missing” mate reads. The presence or absence of other categories distinguishes each type of rearrangement from the others.

Variation in genome structure in the form of rearrangements, duplications, insertions, or deletions of stretches of DNA can also be explored via fragment recruitment. The use of mated sequencing reads (pairs of reads from opposite ends of a clone insert) provides a powerful tool for assessing structural differences between the reference and the environmental sequences. The cloning and sequencing process determines the orientation and approximate distance between two mated sequencing reads. Genomic structural variation can be inferred when these are at odds with the way in which the reads are recruited to a reference sequence. Relative location and orientation of mated sequences provide a form of metadata that can be used to color-code a fragment recruitment plot (Figure 4). This makes it possible to visually identify and classify structural differences and similarities between the reference and the environmental sequences (Figure 5). For the abundant marine microbes, a high proportion of mated reads in the “good” category (i.e., in the proper orientation and at the correct distance) show that synteny is conserved for a large portion of the microbial population. The strongest signals of structural differences typically reflect a variant specific to the reference genome and not found in the environmental data. In conjunction with the requirement that reads be recruited over their entire length without interruption, recruitment plots result in pronounced recruitment gaps at locations where there is a break in synteny. Other rearrangements can be partially present or penetrant in the environmental data and thus may not generate obvious recruitment gaps. However, given sufficient coverage, breaks in synteny should be clearly identifiable using the recruitment metadata based on the presence of “missing” mates (i.e., the mated sequencing read that was recruited but whose mate failed to recruit; Figure 4). The ratio of missing mates to “good” mates determines how penetrant the rearrangement is in the environmental population.

In theory, all genome structure variations that are large enough to prevent recruitment can be detected, and all such rearrangements will be associated with missing mates. Depending on the type of rearrangement present other recruitment metadata categories will be present near the rearrangements' endpoints. This makes it possible to distinguish among insertions, deletions, translocations, inversions, and inverted translocations directly from the recruitment plots. Examples of the patterns associated with different rearrangements are presented in Figure 5. This provides a rapid and easy visual method for exploring structural variation between natural populations and sequenced representatives (Poster S1A and S1B).

### Genomic Structural Variation in Abundant Marine Microbes

Variation in genome structure potentially results in functional differences. Of particular interest are those differences between sequenced (reference) microbes and environmental populations. These differences can indicate how representative a cultivated microbe might be and shed light on the evolutionary forces driving change in microbial populations. Fragment recruitment in conjunction with the mate metadata helped us to identify both the consistent and the rare structural differences between the genomes of microbial populations in the GOS data and their closest sequenced relatives. Our analysis has thus far been confined to the three microbial genera that were widespread in the GOS dataset as represented by the finished genomes of P. marinus MIT9312, P. ubique HTCC1062, and to a lesser extent *Synechococcus* WH8102. Each of these genomes is characterized by large and small segments where little or no fragment recruitment took place. We refer to these segments as “gaps.” These gaps represent reference-specific differences that are not found in the environmental populations rather than a cloning bias that identifies genes or gene segments that are toxic or unclonable in *E. coli.* The presence of missing mates flanking these gaps indicates that the associated clones do exist, and therefore that cloning issues are not a viable explanation for the absence of recruited reads. Although the reference-specific differences are quite apparent due to the recruitment gaps they generate, there are also sporadic rearrangements associated with single clones, mostly resulting from small insertions or deletions.

Careful examination of the unrecruited mates of the reads flanking the gaps allowed us to identify, characterize, and quantify specific differences between the reference genome and their environmental relatives. The results of this analysis for P. ubique and P. marinus have been summarized in Table 5. With few exceptions, small gaps resulted from the insertion or deletion of only a few genes. Many of the genes associated with these small insertions and deletions have no annotated function. In some cases the insertions display a degree of variability such that different sets of genes are found at these locations within a portion of the population. In contrast, many of the larger gaps are extremely variable to the extent that every clone contains a completely unrelated or highly divergent sequence when compared to the reference or to other clones associated with that gap. These segments are hypervariable and change much more rapidly than would be expected given the variation in the rest of the genome. Sites containing a hypervariable segment nearly always contained some insert. We identified two exceptions both associated with *P. ubique.* The first is approximately located at the 166-kb position in the P. ubique HTCC1062 genome. Though no large gap is present, the mated reads indicate that under many circumstances a highly variable insert is often present. The second is a gap on HTCC1062 that appears between 50 and 90 kb. This gap appears to be less variable than other hypervariable segments and is occasionally absent based on the large numbers of flanking long mated reads (Poster S1A). Interestingly, the long mated reads around this gap seem to be disproportionately from the Sargasso Sea samples, suggesting that this segment may be linked to geographic and/or environmental factors. Thus, hypervariable segments are highly variable even within the same sample, can on occasion be unoccupied, and the variation, or lack thereof, can be sample dependent.

**Table 5:**
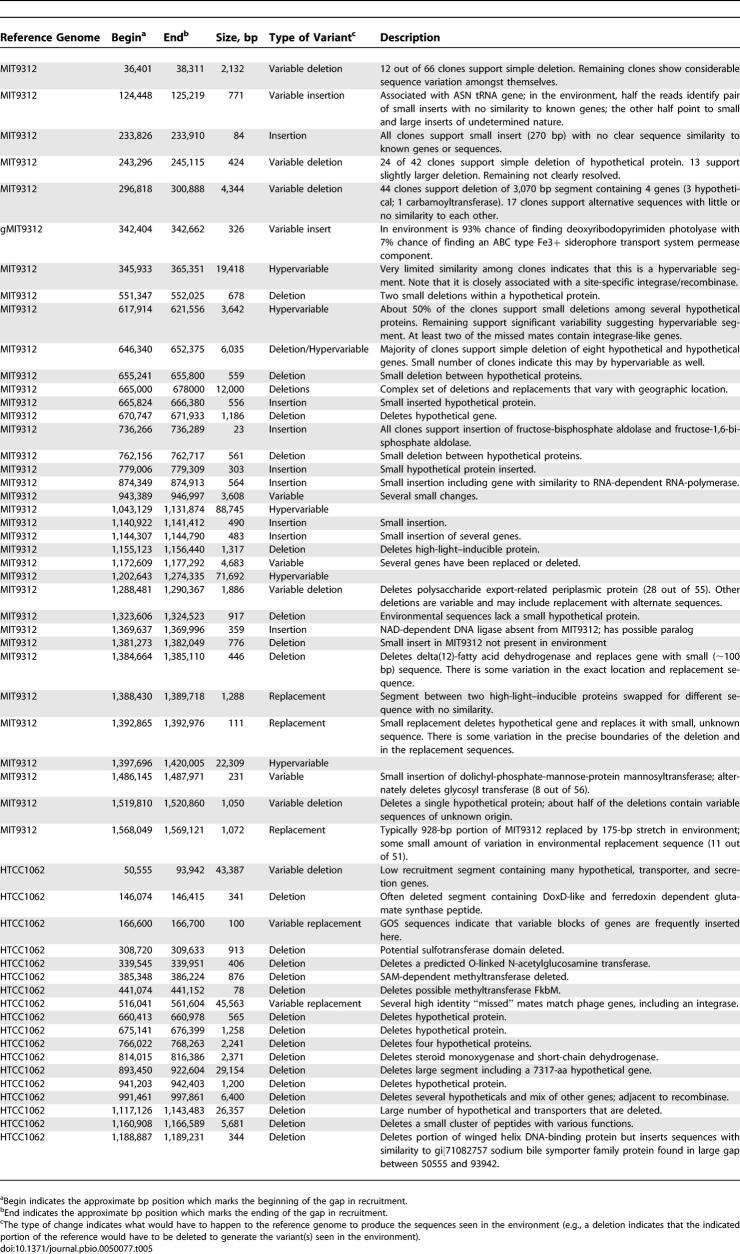
Atypical Segments in P. marinus MIT9312 and P. ubique HTCC1062 (SAR11)

Hypervariable segments have been seen previously in a wide range of microbes, including P. marinus [28], but their precise source and functional role, especially in an environmental context, remains a matter of ongoing research. For clues to these issues we examined the genes associated with the missing mates flanking these segments and the nucleotide composition of the gapped sequences in the reference genomes. In some rare cases the genes identified on reads that should have recruited within a hypervariable gap were highly similar to known viral genes. For example, a viral integrase was associated with the P. ubique HTCC1062 hypervariable gap between 516 and 561 kb. However, in the majority of cases the genes associated with these gaps were uncharacterized, either bearing no similarity to known genes or resembling genes of unknown function. If these genes were indeed acquired through horizontal transfer then we might expect that they would have obvious compositional biases. Oligonucleotide frequencies along the P. ubique HTCC1062 and *Synechococcus* WH8102 genomes are quite different in the large recruitment gaps in comparison to the well-represented portions of the genome (Poster S1). Surprisingly, this was less true for P. marinus MIT9312, where the gaps have been linked to phage activity [28]. These results suggest that these hypervariable segments of the genome are widespread among marine microbial populations, and that they are the product of horizontal transfer events perhaps mediated by phage or transposable elements. These results are consistent with and expand upon the hypothesis put forward by Coleman et al. [28] suggesting that these segments are phage mediated, and conflicts with initial claims that the HTCC1062 genome was devoid of genes acquired by horizontal transfer [29].

Though insertions and deletions accounted for many of the obvious regions of structural variation, we also looked for rearrangements. The high levels of local synteny associated with P. ubique and P. marinus suggested that large-scale rearrangements were rare in these populations. To investigate this hypothesis we used the recruitment data to examine how frequently rearrangements besides insertions and deletions could be identified. We looked for rearrangements consisting of large (greater than 50 kb) inversions and translocations associated with *P. marinus;* however, we did not identify any such rearrangements that consistently distinguished environmental populations from sequenced cultivars. Rare inversions and translocations were identified in the dominant subtype associated with MIT9312 (Table 6). Based on the amount of sequence that contributed to the analysis, we estimate that one inversion or translocation will be observed for every 2.6 Mbp of sequence examined (less than once per P. marinus genome).

**Table 6:**
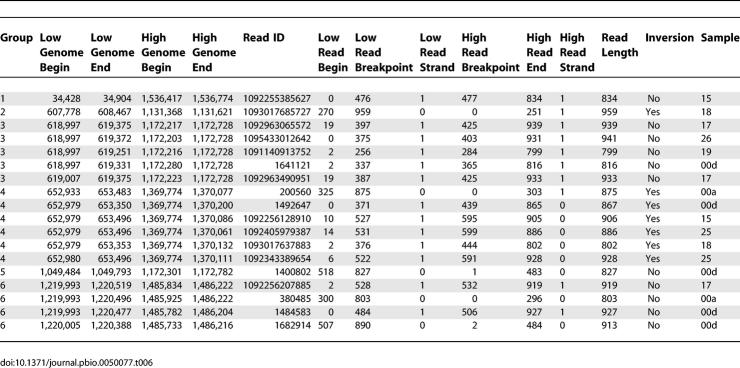
Six Large-Scale Translocations and Inversions Were Identified in the Abundant P. marinus Subtype

A further observation concerns the uniformity along a genome of the evolutionary history among and within subtypes. For instance, the similarity between GOS reads and P. marinus MIT9312 is typically 85%–95%, while the similarity between MIT9312 and P. marinus MED4 is generally ~10% lower. However, there are several instances where the divergence of MIT9312 and MED4 abruptly decreases to no more than that between the GOS sequences and MIT9312 (Poster S1G). These results are consistent either with horizontal transfer (recombination) or with inhomogeneous selectional pressures. Similar patterns are present in the two high-identity subtypes seen on the P. ubique HTCC1062 genome (Poster S1D). Other regions show local increases in similarity between MIT9312 and the dominant subtype that are not reflected in the MIT9312/MED4 divergence (e.g., near positions 50 kb, 288 kb, 730 kb, 850 kb, and 954 kb on MIT9312; also see Poster S1G). These latter regions might reflect either regions of homogenizing recombination or regions of higher levels of purifying selection. However, the lengths of the intervals (several are 10 kb or more) are longer than any single gene and correspond to genes that are not extremely conserved over greater taxonomic distances (in contrast to the ribosomal RNA operon). Equally, if widespread horizontal transfer of an advantageous segment explains these intervals, the transfers occurred long enough ago for appreciable variation to accumulate (unpublished data).

### Extreme Assembly of Uncultivated Populations

**Figure 6:**
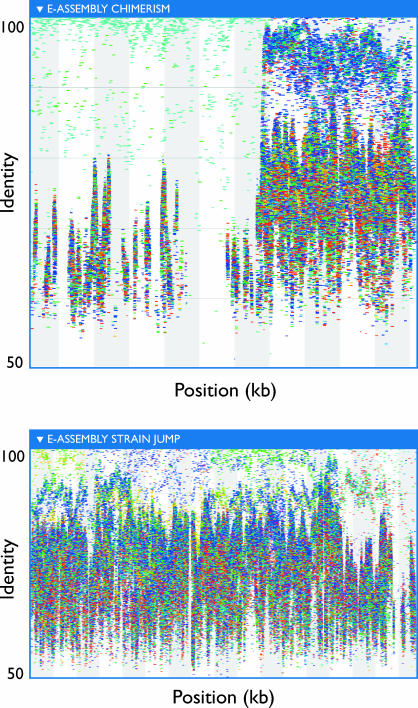
Examples of Chimeric Extreme Assemblies (A) Fragment recruitment to an extreme assembly contig indicates the assembly is chimeric between two organisms, based on dramatic shifts in density of recruitment, level of conservation, and sample distribution. (B) Fragment recruitment to a SAR11-related extreme assembly. Changes in color, density, and vertical location toward the top of the figure indicate transitions among multiple subtypes of SAR11.

The analyses described above have been confined to those organisms with representatives in culture and for which genomes were readily available. Producing assemblies for other abundant but uncultivated microbial genera would provide valuable physiological and biochemical information that could eventually lead to the cultivation of these organisms, help elucidate their role in the marine community, and allow similar analyses of their evolution and variation such as those performed on sequenced organisms. Previous assembly efforts and the fragment recruitments plots showed that there is considerable and in many cases conflicting variation among related organisms. Such variation is known to disrupt whole-genome assemblers. This led us to try an assembly approach that aggressively resolves conflicts. We call this approach “extreme assembly” (see Materials and Methods). This approach currently does not make use of mate-pairing data and, therefore produces only contigs, not scaffolded sequences. Using this approach, contigs as large as 900 kb could be aligned almost in their entirety to the P. marinus MIT9312 and P. ubique HTCC1062 genomes (Figure 2J–2L). Consistent patterns of fragment recruitment (see below) generally provided evidence of the correctness of contigs belonging to otherwise-unsequenced organisms. Accordingly, large contigs from these alternate assemblies were used to investigate genetic and geographic population structure, as described below. However, the more aggressive assemblies demonstrably suffered from higher rates of assembly artifacts, including chimerism and false consensus sequences (Figure 6). Thus, the more stringent primary assembly was employed for most assembly-based analyses, as manual curation was not practical.

As just noted, many of the large contigs produced by the more aggressive assembly methods described above did not align to any great degree with known genomes. Some could be tentatively classified based on contained 16S sequences, but the potential for computationally generated chimerism within the rRNA operon is sufficiently high that inspection of the assembly or other means of confirming such classifications is essential. An alternative to an unguided assembly that facilitates the association of assemblies with known organisms is to start from seed fragments that can be identified as belonging to a particular taxonomic group. We employed fragments outside the ribosomal RNA operon that were mated to a 16S-containing read, limiting extension to the direction away from the 16S operon. This produced contigs of 100 kb or more for several of the ribotypes that were abundant in the GOS dataset. When evaluated via fragment recruitment (Figure 2M–2O), these assemblies revealed patterns analogous to those seen for the sequenced genomes described above: multiple subtypes could be distinguished along the assembly, differing in similarity to the reference sequence and sample distribution, with occasional gaps. Hypervariable segments by definition were not represented in these assemblies, but they may help explain the termination of the extreme assemblies for P. marinus and SAR11 and provide a plausible explanation for termination of assemblies of the other deeply sampled populations as well.

**Figure 7:**
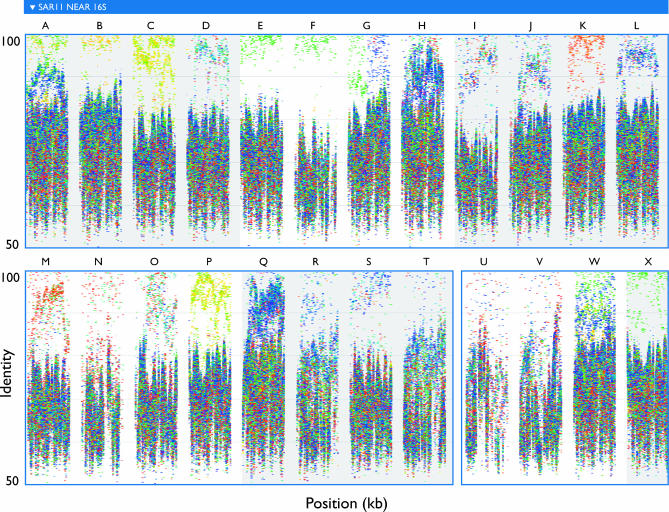
Fragment Recruitment Plots to 20-kb Segments of SAR11-Like Contigs Show That Many SAR11 Subtypes, with Distinct Distributions, Can Be Separated by Extreme Assembly Each segment is constructed of a unique set of GOS sequencing reads (i.e., no read was used in more than one segment). Segments are arbitrarily labeled (A–X) for reference in Figure 8.

**Figure 8:**
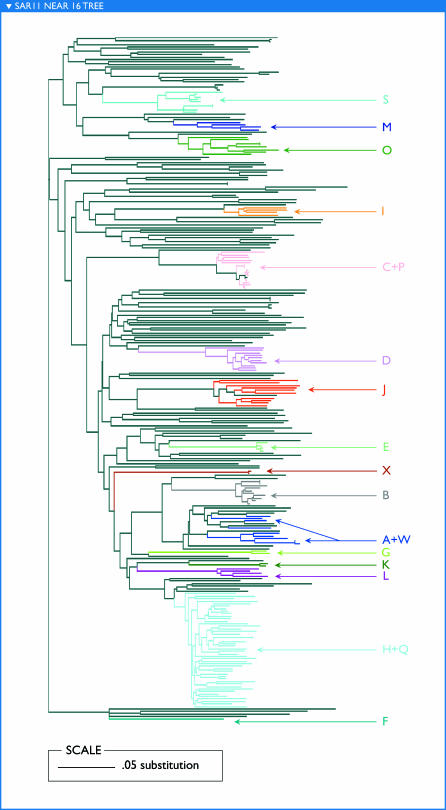
Phylogeny of GOS Reads Aligning to P. ubique HTCC1062 Upstream of 16S Gene Indicates That the Extreme Assemblies in Figure 7 Correspond to Monophyletic Subtypes Coloring of branches indicates that the corresponding reads align at >90% identity to the extreme assembly segments shown in Figure 7; colored labels (A–X) correspond to the labels in Figure 7, indicating the segment or segments to which reads aligned.

This directed approach to assembly can also be used to investigate variation within a group of related organisms (e.g., a 16S ribotype). We explored the potential to assemble distinct subtypes of SAR11 by repeatedly seeding extreme assembly with fragments mated to a SAR11-like 16S sequence. Figure 7 compares the first 20 kb from each of 24 independent assemblies. Eighteen of these segments could be aligned full-length to a portion of the HTCC1062 genome just upstream of 16S, while six appeared to reflect rearrangements relative to HTCC1062. The rearranged segments were associated with more divergent 16S sequences (8%–14% diverged from the 16S of HTCC1062), while those without rearrangements corresponded to less divergent 16S (averaging less than 3% different from HTCC1062). In each segment, many reads were recruited above 90% identity, but different samples dominated different assemblies. Phylogenetic trees support the inference of evolutionarily distinct subtypes with distinctive sample distributions (Figure 8).

### Taxonomic Diversity

Environmental surveys provide a cultivation-independent means to examine the diversity and complexity of an environmental sample and serve as a basis to compare the populations between different samples. Typically, these surveys use PCR to amplify ubiquitous but slowly evolving genes such as the 16S rRNA or *recA* genes. These in turn can be used to distinguish microbial populations. Since PCR can introduce various biases, we identified 16S genes directly from the primary GOS assembly. In total, 4,125 distinct full-length or partial 16S were identified. Clustering of these sequences at 97% identity gave a total of 811 distinct ribotypes. Nearly half (48%) of the GOS ribotypes and 88% of the GOS 16S sequences were assigned to ribotypes previously deposited in public databases. That is, more than half the ribotypes in the GOS dataset were found to be novel at what is typically considered the species level [30]. The overall taxonomic distribution of the GOS ribotypes sampled by shotgun sequencing is consistent with previously published PCR based studies of marine environments (Table 7) [31]. A smaller amount (16%) of GOS ribotypes and 3.4% of the GOS 16S sequences diverged by more than 10% from any publicly available 16S sequence, thus being novel to at least the family level.

**Table 7:**
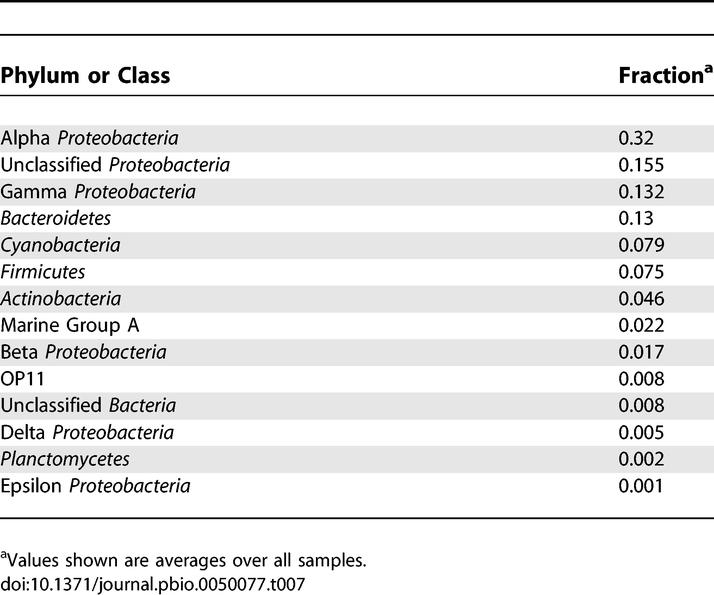
Taxonomic Makeup of GOS Samples Based on 16S Data from Shotgun Sequencing

A census of microbial ribotypes allows us to identify the abundant microbial lineages and estimate their contribution to the GOS dataset. Of the 811 ribotypes, 60 contain more than 8-fold coverage of the 16S gene (Table 8); jointly, these 60 ribotypes accounted for 73% of all the 16S sequence data. All but one of the 60 have been detected previously, yet only a few are represented by close relatives with complete or nearly complete genome sequencing projects (see Fragment Recruitment for further details). Several other abundant 16S sequences belong to well-known environmental ribotypes that do not have cultivated representatives (e.g., SAR86, *Roseobacter* NAC-1–2, and branches of SAR11 other than those containing P. ubique). Interestingly, archaea are nearly absent from the list of dominant organisms in these near-surface samples.

**Table 8:**
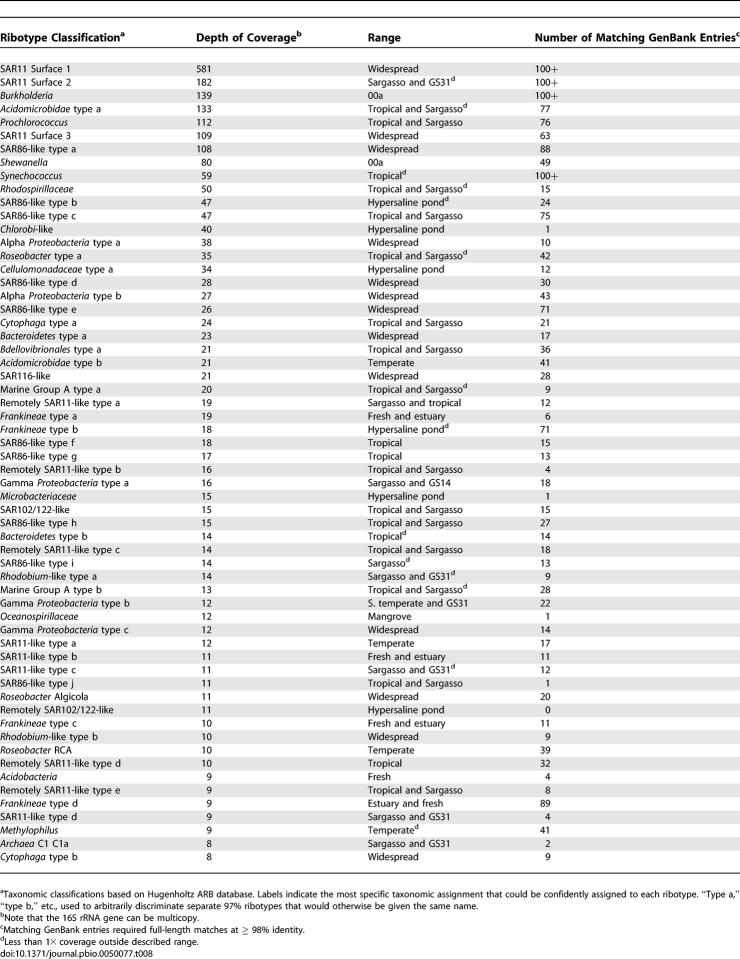
Most Abundant Ribotypes (97% Identity Clusters)

**Figure 9:**
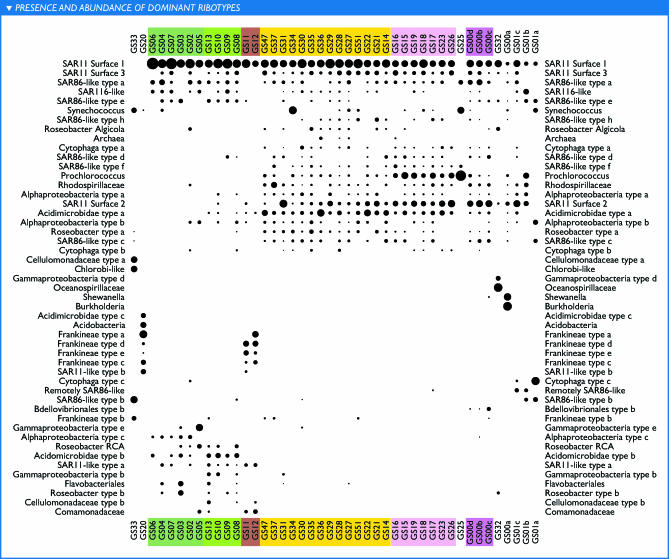
Presence and Abundance of Dominant Ribotypes The relative abundance of various ribotypes (rows) in each filter (columns) is represented by the area of the corresponding spot (if any). The listed ribotypes each satisfied the following criteria in at least one filter: the ribotype was among the five most abundant ribotypes detected in the shotgun data, and was represented by at least three sequencing reads. Relative abundance is based on the total number of 16S sequences in a given filter. Order and grouping of filters is based on the clustering of genomic similarity shown in Figure 11. Ribotype order was determined based on similarity of sample distribution. A marked contrast between temperate and tropical groups is visible. Estuarine samples GS11 and GS12 contained a mix of ribotypes seen in freshwater and temperate marine samples, while samples from nonmarine habitats or larger filter sizes were pronounced outliers. The presence of large amounts of *Burkholderia* and *Shewanella* in one Sargasso Sea sample (GS00a) makes this sample look much less like other Sargasso and tropical marine samples than it otherwise would. Note that 16S is not a measure of cell abundance since 16S genes can be multicopy.

The distribution of these ribotypes reveals distinct microbial communities (Figure 9 and Table 8). Only a handful of the ribotypes appear to be ubiquitously abundant; these are dominated by relatives of SAR11 and SAR86. Many of the ribotypes that are dominant in one or more samples appear to reside in one of three separable marine surface habitats. For example, several SAR11, SAR86, and alpha *Proteobacteria,* as well as an *Acidimicrobidae* group, are widespread in the surface waters, while a second niche delineated by tropical samples contains several different SAR86, *Synechococcus* and *Prochlorococcus* (both cyanobacterial groups), and a *Rhodospirillaceae* group. Other ribotypes related to *Roseobacter* RCA, SAR11, and gamma *Proteobacteria* are abundant in the temperate samples but were not observed in the tropical or Sargasso samples. Not surprisingly, samples taken from nonmarine environments (GS33, GS20, GS32), estuaries (GS11, GS12), and larger-sized fraction filters (GS01a, GS01b, GS25) have distinguishing ribotypes. Furthermore, as the complete genomes of these dominant members are obtained, the capabilities responsible for their abundances may well lend insight into the community metabolism in various oceanic niches.

**Figure 10:**
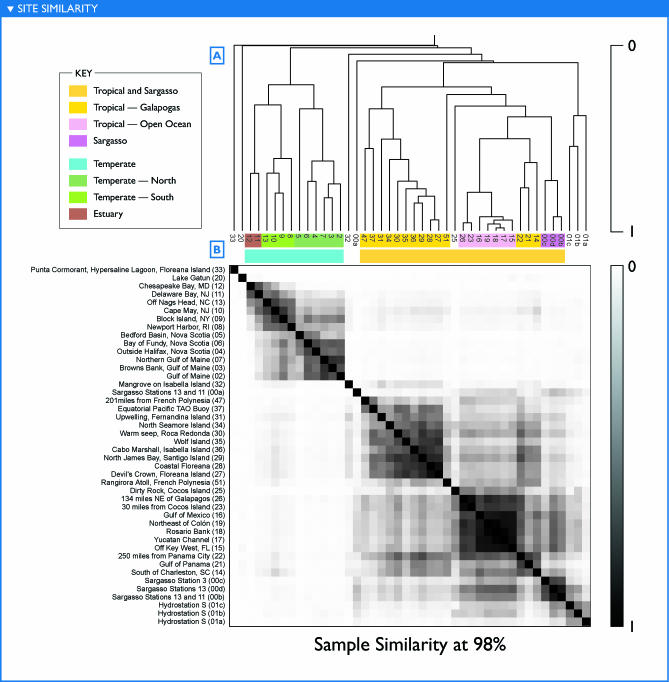
Similarity between Samples in Terms of Shared Genomic Content Genomic similarity, as described in the text, is an estimate of the amount of the genetic material in two filters that is “the same” at a given percent identity cutoff—not the amount of sequence in common in a finite dataset, but rather in the total set of organisms present on each filter. Similarities are shown for 98% identity. (A) Hierarchical clustering of samples based on pairwise similarities. (B) Pairwise similarities between samples, represented as a symmetric matrix of grayscale intensities; a darker cell in the matrix indicates greater similarity between the samples corresponding to the row and column, with row and column ordering as in (A). Groupings of similar filters appear as subtrees in (A) and as squares consisting of two or more adjacent rows and columns with darker shading. Colored bars highlight groups of samples described in the text; labels are approximate characterizations rather than being strictly true of every sample in a group.

**Figure 11:**
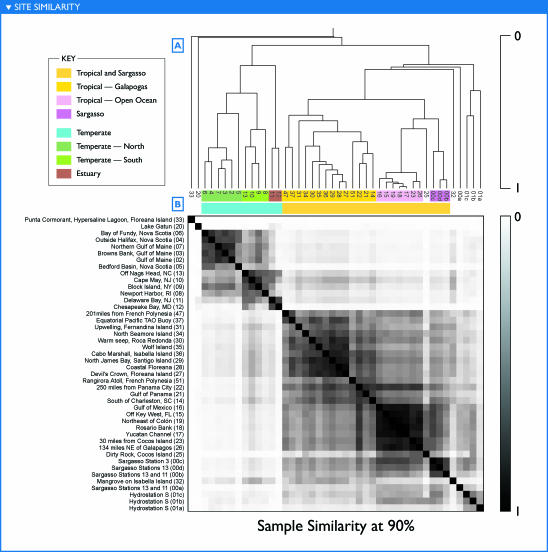
Sample Similarity at 90% Identity Similarity between samples in terms of shared genomic content similar to Figure 10, except that the plots were done using a 90% identity cutoff that has proven reasonable for separating some moderately diverged subtypes

### Sample Comparisons

The most common approach for comparing the microbial community composition across samples has been to examine the ribotypes present as indicated by 16S rRNA genes or by analyzing the less-conserved ITS located between the 16S and 23S gene sequences [7,8,16,17]. However, a clear observation emerging from the fragment recruitment views was that the reference ribotypes recruit multiple subtypes, and that these subtypes were distributed unequally among samples (Figures 2, 7, 8; Poster S1D, S1F, and S1I).

We developed a method to assess the genetic similarity between two samples that potentially makes use of all portions of a genome, not just the 16S rRNA region. This similarity measure is assembly independent; under certain circumstances, it is equivalent to an estimate of the fraction of sequence from one sample that could be considered to be in the other sample. Whole-metagenomic similarities were computed for all pairs of samples. Results are presented for comparisons at ≥98% and 90% identity. No universal cutoff consistently divides sequences into natural subsets, but the 98% identity cutoff provides a relatively high degree of resolution, while the 90% cutoff appears to be a reasonable heuristic for defining subtypes. For instance, a 90% cutoff treats most of the reads specifically recruited to P. marinus MIT9312 as similar (those more similar to MED4 notably excepted), while reasonably separating clades of SAR11 (Figures 7 and 8). Reads with no qualifying overlap alignment to any other read in a pair of samples are uninformative for this analysis, as they correspond to lineages that were so lightly sequenced that their presence in one sample and absence in another may be a matter of chance. For the 90% cutoff, 38% of the sequence reads contributed to the analysis. The resulting similarities reveal clear and consistent groupings of samples, as well as the outlier status of certain samples (Figures 10 and 11).

The broadest contrast was between samples that could be loosely labeled “tropical” (including samples from the Sargasso Sea [GS00b, GS00c, GS00d] and samples that are temperate by the formal definition but under the influence of the Gulf Stream [GS14, GS15]) and “temperate.” Further subgroups can be identified within each of these categories, as indicated in Figures 10 and 11. In some cases, these groupings were composed of samples taken from different ocean basins during different legs of the expedition. A few pairs of samples with strikingly high similarity were observed, including GS17 and GS18, GS23 and GS26, GS27 and GS28, and GS00b and GS00d. In each case, these pairs of samples were collected from consecutive or nearly consecutive samples. However, the same could be said of many other pairs of samples that do not show this same degree of similarity. Indeed, geographically and temporally separated samples taken in the Atlantic (GS17, GS18) and Pacific (GS23, GS26) during separate legs of the expedition are more similar to one another than were most pairs of consecutive samples. The samples with least similarity to any other sample were from unique habitats. Thus, similarity cannot be attributed to geographic separation alone.

The groupings described above can be reconstructed from taxonomically distinct subsets of the data. Specifically, the major groups of samples visible in Figure 10 were reproduced when sample similarities were determined based only on fragments recruiting to P. ubique HTCC1062 (unpublished data). Likewise, the same groupings were observed when the fragments recruiting to either HTCC1062 or P. marinus MIT9312, or both, were excluded from the calculations (unpublished data). Thus, the factors influencing sample similarities do not appear to rely solely on the most abundant organisms but rather are reflected in multiple microbial lineages.

It is tempting to view the groups of similar samples as constituting community types. Sample similarities based on genomic sequences correlated significantly with differences in the environmental parameters (Table 1), particularly water temperature and salinity (unpublished data). Samples that are very similar to each other had relatively small differences in temperature and salinity. However, not all samples that had similar temperature and salinity had high community similarities. Water depth, primary productivity, fresh water input, proximity to land, and filter size appeared consistent with the observed groupings. Other factors such as nutrients and light for phototrophs and fixed carbon/energy for chemotrophs may ultimately prove better predictors, but these results demonstrate the potential of using metagenomic data to tease out such relationships.

Examining the groupings in Figure 11 in light of habitat and physical characteristics, the following may be observed. The first two samples, a hypersaline pond in the Galapagos Islands (GS33) and the freshwater Lake Gatun in the Panama Canal (GS20) are quite distinct from the rest. Salinity—both higher and lower than the remaining coastal and ocean samples—is the simplest explanation.

Twelve samples form a strong temperate cluster as seen in the similarity matrix of Figure 11 as a darker square bounded by GS06 and GS12. Embedded within the temperate cluster are three subclusters. The first subcluster includes five samples from Nova Scotia through the Gulf of Maine. This is followed by a subcluster of four samples between Rhode Island and North Carolina. The northern subcluster was sampled in August, the southern subcluster in November and December. Though all samples were collected in the top few meters, the southern samples were in shallower waters, 10 to 30 m deep, whereas most of the northern samples were in waters greater than 100 m deep. Monthly average estimates of chlorophyll *a* concentrations were typically higher in the southern samples as well (Table 1). All of these factors—temperature, system primary production, and depth of the sampled water body—likely contribute to the differences in microbial community composition that result in the two well-defined clusters. The final temperate subgroup includes two estuaries, Chesapeake Bay (GS12) and Delaware Bay (GS11), distinguished by their lower salinity and higher productivity. However, GS11 is markedly similar not only to GS12 but also to coastal samples, whereas the latter appears much more unique. Interestingly, the Bay of Fundy estuary sample (GS06) clearly did not group with the two other estuaries, but rather with the northern subgroup, perhaps reflecting differences in the rate or degree of mixing at the sampling site.

Continuing to the right and downward in Figure 11, one can see a large cluster of 25 samples from the tropics and Sargasso Sea, bounded by GS47 and GS00b. This can be further subdivided into several subclusters. The first subcluster (a square bounded by GS47 and GS14) includes 14 samples, about half of which were from the Galapagos. The second distinct subcluster (a square bounded by GS16 and GS26) includes seven samples from Key West, Florida, in the Atlantic Ocean to a sample close to the Galapagos Islands in the Pacific Ocean. Loosely associated with this subcluster is a sample from a larger filter size taken en route to the Galapagos (GS25). The remaining samples group weakly with the tropical cluster. GS32 was taken in a coastal mangrove in the Galapagos. The thick organic sediment at a depth of less than a meter is the likely cause for it being unlike the other samples. Sample 00a was from the Sargasso Sea and contained a large fraction of sequence reads from apparently clonal *Burkholderia* and *Shewanella* species that are atypical. When this sample is reanalyzed to exclude reads identified as belonging to these two groups, sample GS00a groups loosely with GS00b, GS00c, and GS00d (unpublished data). Finally, three subsamples from a single Sargasso sample (GS01a, GS01b, GS01c) group together, despite representing three distinct size fractions (3.0–20, 0.8–3.0, and 0.1–0.8 μm, respectively; Table 1).

The complete set of sample similarities is more complex than described above, and indeed is more complex than can be captured by a hierarchical clustering. For instance, the southern temperate samples are appreciably more similar to the tropical cluster than are the northern temperate samples. GS22 appears to constitute a mix of tropical types, showing strong similarity not only to the GS47–GS14 subcluster to which it was assigned, but also to the other tropical samples.

These results may be compared to the more traditional view of community structure afforded by 16S sequences (Figure 9). Some of the same groupings of samples are visible using both analyses. Several ribotypes recapitulated the temperate/tropical clustering described above. Others were restricted to the single instances of nonmarine habitats. Several of the most abundant organisms from the coastal mangrove, hypersaline lagoon, and freshwater lake were found exclusively in these respective samples. However, while several ribotypes recapitulated the temperate/tropical distinction revealed by the genomic sequence, others crosscut it. A few dominant 16S ribotypes, related to SAR11, SAR86, and SAR116, were found in every marine sample. The brackish waters from two mid-Atlantic estuaries (GS11 and GS12) contained a mixture of otherwise exclusively marine and freshwater ribotypes; similarity of these sites to the freshwater sample (GS20) was minimal at the metagenomic level, while the greater similarity of GS11 to coastal samples visible at the metagenomic level was not readily visible here. A fuller comparison of metagenome-based measurements of diversity based on a large dataset of PCR-derived 16S sequences will be presented in another paper (in preparation).

### Variation in Gene Abundance

Differences in gene content between samples can identify functions that reflect the lifestyles of the community in the context of its local environment [20,32]. We examined the relative abundance of genes belonging to specific functional categories in the distinct GOS samples. Genes were binned into functional categories using TIGRFAM hidden Markov models [18], which are well annotated and manually curated [33].

The results can be filtered in various ways to highlight genes associated with specific environments. One catalog of possible interest is genes that were predominantly found in a single sample. We identified 95 TIGRFAMs that annotated large sets of genes (100 or more) that were significantly more frequent (greater than 2-fold) in one sample than in any other sample (Table 9). Not surprisingly, this approach disproportionately singles out genes from the samples collected on larger filters (GS01a, GS01b, and GS25) and from the nonmarine environments, particularly the hypersaline pond (sample GS33). Another contrast might be between the temperate and tropical clusters (Figures 10 and 11). We identified 32 proteins that were more than 2-fold more frequent in one or the other group (Table 10). The presence of various *Prochlorococcus*-associated genes in this list highlights some of the potential challenges with this sort of approach. Overrepresentation may reflect: a direct response to particular environmental pressures (as the excess of salt transporters plausibly do in the hypersaline pond); a lineage-restricted difference in functional repertoire (as exemplified by the excess of photosynthesis genes in samples containing *Prochlorococcus*); or a more incidental “hitchhiking” of a protein found in a single organism that happens to be present.

**Table 9:**
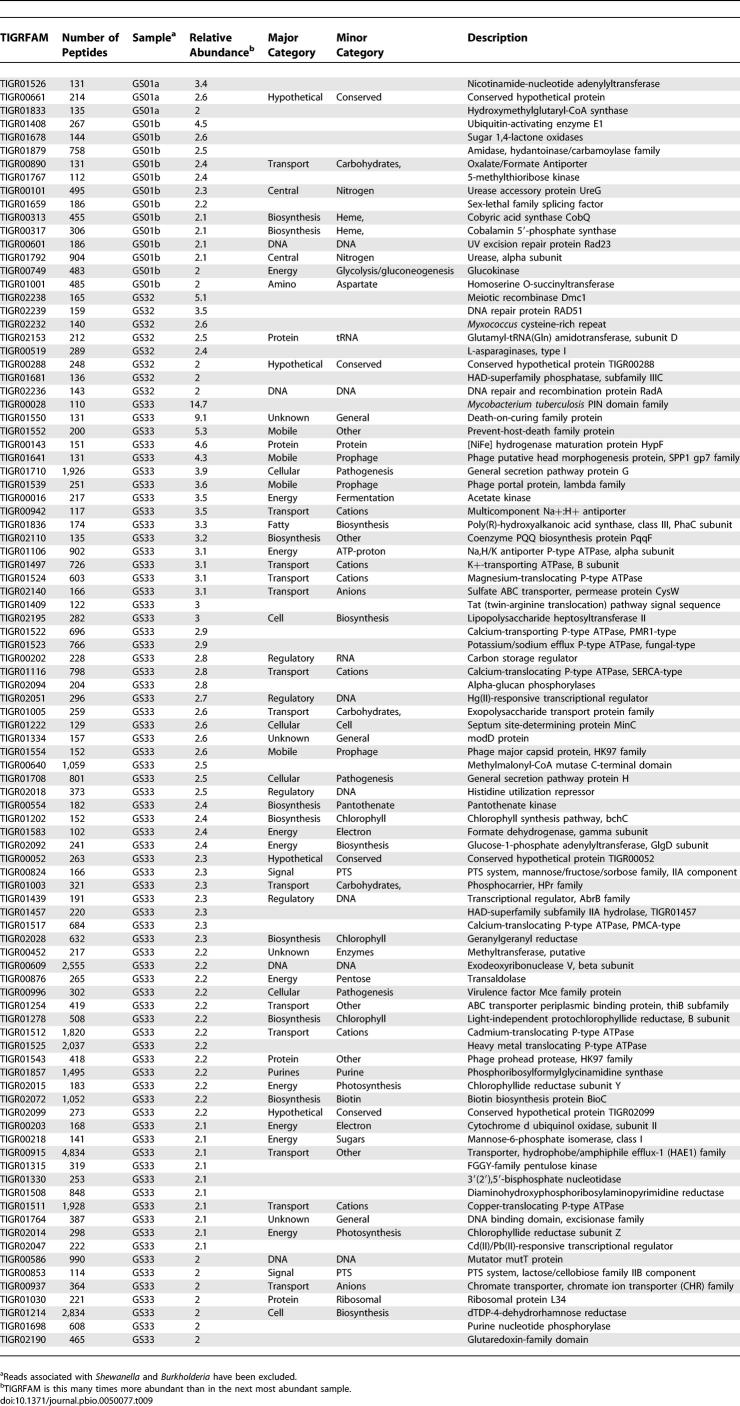
Relative Abundance of TIGRFAMs Associated with a Specific Sample

**Table 10:**
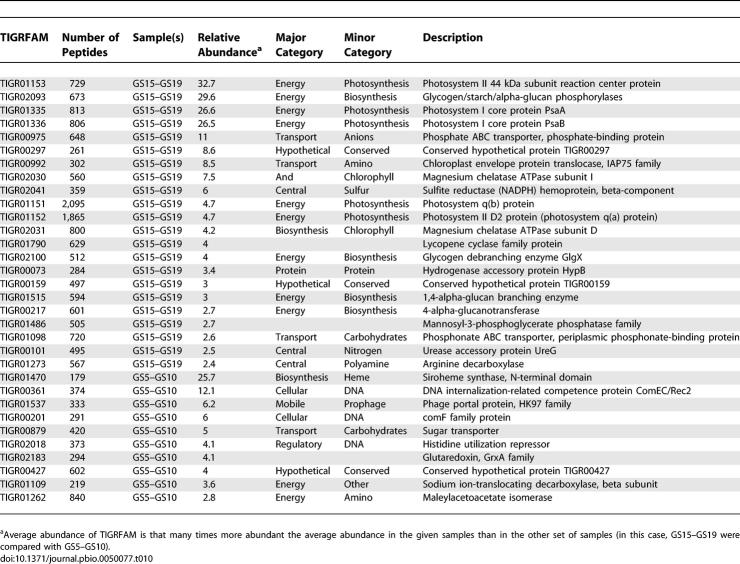
Relative Abundance of TIGRFAM Matches in Temperate and Tropical Waters

We explored whether clearer and more informative differences could be discovered between communities by focusing on groups of samples that are highly similar in overall taxonomic/genetic content. Two pairs of samples provide a particularly nice illustration of this approach. Samples GS17 and GS18 from the western Caribbean Sea and samples GS23 and GS26 from the eastern Pacific Ocean were all very similar based on the presence of abundant ribotypes and overall similarity in genetic content (Figures 9–11). Despite these similarities, several genes are found to be up to seven times more common in the pair of Caribbean samples than the Pacific pair (Table 11). No genes are more than 2-fold higher in the Pacific than the Caribbean pair of samples. Several of the most differentially abundant genes are related to phosphate transport and utilization. It is very plausible that this is a reflection of a functional adaptation: these differences correlate well with measured differences in phosphate abundance between the Atlantic and eastern Pacific samples [34,35], and phosphate abundance plays a critical role in microbial growth [36,37]. Indeed, the ability to acquire phosphate, especially under conditions where it is limited, is thought to determine the relative fitness of *Prochlorococcus* strains [38].

**Table 11:**
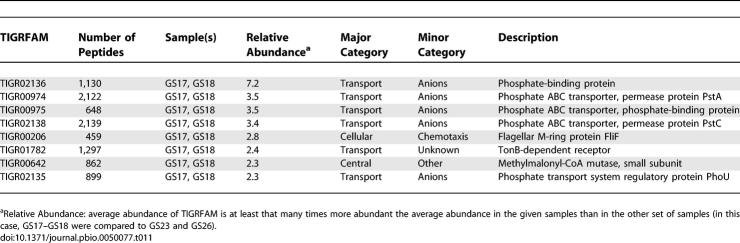
Relative Abundance of TIGRFAM Matches in Atlantic and Pacific Open Ocean Waters

The single greatest difference between GS17 and GS18 on the one hand and GS23 and GS26 on the other was attributed to a set of genes annotated by the hidden Markov model TIGR02136 as a phosphate-binding protein *(PstS).* This TIGRFAM identified a single gene in both P. marinus MIT9312 and P. ubique HTCC1062. In P. marinus MIT9312, this gene is located at 672 kb lying roughly in the middle of a 15-kb segment of the genome that recruits almost no GOS sequences from the Pacific sampling sites (Poster S1H). In P. ubique HTCC1062, the *PstS* gene is found at 1,133 kb in a 5-kb segment that also recruited far fewer GOS sequences from all the Pacific samples except for GS51 (Poster S1E). These genomic segments differ structurally among isolates but they are no more variable than the flanking regions, and thus are not hypervariable in the sense used previously (unpublished data). Nor are they particularly conserved when present, indicating that they are not the result of a recent lateral transfer. Phylogenetic analyses outside these segments did not produce any evidence of a Pacific versus Caribbean clade of either *Prochlorococcus* or SAR11 (Figure 3A–3B). The presence or absence of phosphate transporters is not limited to these two types of organisms. The number of phosphate transporters that were found in the Caribbean far exceeds the number that can be attributed to HTCC1062- and MIT9312-like organisms. However, these results indicate that within individual strains or subtypes the ability to acquire phosphate (in one or more of its forms) can vary without detectable differences in the surrounding genomic sequences.

### Biogeographic Distribution of Proteorhodopsin Variants

**Figure 12:**
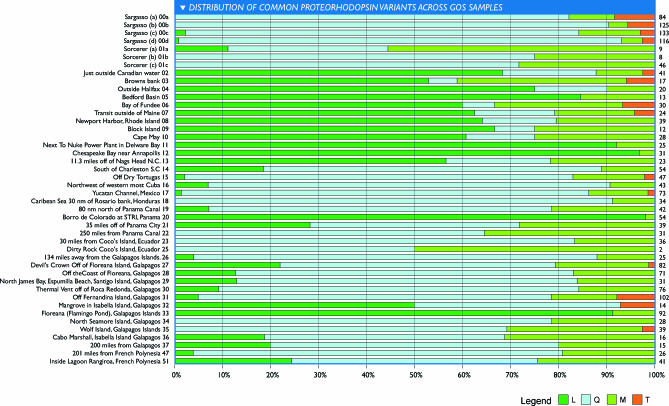
Distribution of Common Proteorhodopsin Variants across GOS Samples The leucine (L) and methionine (M) variants absorb maximally in the green spectrum (Oded Beja, personal communication) while the glutamine (Q) variant absorbs maximally in the blue spectrum. The relative abundance of each variant is shown as a percentage (*x*-axis) per sample (*y*-axis). Total abundance for all variants in read equivalents normalized by the abundance of recA protein are shown on the right side of the *y*-axis. The L and Q variants show a nonrandom distribution. The L variant is abundant in temperate Atlantic waters close to the U.S. and Canadian coast. The Q variant is abundant in warmer waters further from land. The M variant is moderately abundant in a wide range of samples with no obvious geographic/environmental association.

Variation in gene content is only one aspect of the tremendous diversity in the GOS data. The functional significance of all the polymorphic differences between homologous proteins remains largely unknown. To look for functional differences, we analyzed members of proteorhodopsin gene family. Proteorhodopsins are fast, light-driven proton pumps for which considerable functional information is available though their biological role remains unknown. Proteorhodopsins were highly abundant in the Sargasso Sea samples [19] and continue to be highly abundant and evenly distributed (relative to *recA* abundance) in all the GOS samples. A total of 2,674 putative proteorhodopsin genes were identified in the GOS dataset. Although many of the sequences are fragmentary, 1,874 of these genes contain the residue that is primarily responsible for tuning the light-absorbing properties of the protein [39–41], and these properties have been shown to be selected for under different environmental conditions [42]. Variation at this residue is strongly correlated with sample of origin (Figure 12). The leucine (L) or green-tuned variant was highly abundant in the North Atlantic samples and in the nonmarine environments like the fresh water sample from Lake Gatun (GS20). The glutamine (Q) or blue-tuned variant dominated in the remaining mostly open ocean samples.

Given our limited understanding of the biological role for proteorhopsin, the reason for this differential distribution is not immediately clear. In coastal waters where nutrients are more abundant, phytoplankton is dominant. Phytoplankton absorbs primarily in the blue and red spectra; consequently, the water appears green [43]. Conversely, in the open ocean nutrients are rare and phytoplanktonic biomass is low, so waters appear blue because in the absence of impurities the red wavelengths are absorbed preferentially [44]. It may be that proteorhodopsin-carrying microbes have simply adapted to take advantage of the most abundant wavelengths of light in these systems.

Proteorhodopsins encoded on reads that were recruited to P. ubique HTCC1062 account for a fraction (~25%) of all the proteorhodopsin-associated reads, suggesting that the remainder must be associated with a variety of marine microbial taxa (see also [45–47]). Phylogenetic analysis of the SAR11-associated proteins revealed that each variant has arisen independently at least two times in the SAR11 lineage (Figure 3C). Consistent with other findings that proteorhodopsins are widely distributed throughout the microbial world [48], we conclude that multiple microbial lineages are responsible for proteorhodopsin spectral variation and that the abundance of a given variant reflects selective pressures rather than taxonomic effects. Similar mechanisms seem to be involved in the evolution and diversification of opsins that mediate color vision in vertebrates [49].

## Discussion

Our results highlight the astounding diversity contained within microbial communities, as revealed through whole-genome shotgun sequencing carried out on a global scale. Much of this microbial diversity is organized around phylogenetically related, geographically dispersed populations we refer to as subtypes. In addition, there is tremendous variation within subtypes, both in the form of sequence variation and in hypervariable genomic islands. Our ability to make these observations derived from not only the large volumes of data but also from the development of new tools and techniques to filter and organize the information in manageable ways.

### Variation and Diversity

Our data demonstrate to an unprecedented degree the nature and evolution of genetic variation below the species level. Variation can be analyzed in several ways, including observed differences in sequence, genomic structure, and gene complement. The observed patterns of variation shed light on the mechanisms by which marine prokaryotes evolve. Gene synteny seems to be more highly conserved than the nucleotide and protein sequences. This variation is seen over essentially the entire genome in every abundant group of organisms sufficiently related for us to recognize a population by fragment recruitment. (These include, but are not limited to, the organisms shown in Figure 2 and Poster S1.) Notably, we found no evidence of widespread low-diversity organisms such as B. anthracis [50].

Phylogenetic trees and fragment recruitment plots (Figures 7 and 8) indicate that the variation within a species is not an unstructured swarm or cloud of variants all equally diverged from one another. Instead, there are clearly distinct subtypes, in terms of sequence similarity, gene content, and sample distribution. Similar findings have been shown for specific organisms, based on evaluation of one or a few loci [2,51–53]. These results rule out certain trivial models of population history and evolution for what is commonly considered a bacterial “species.” For instance, it argues against a recent explosive population growth from a single successful individual (selective sweep) [54]. Equally, it argues against a perfectly mixed population, suggesting instead some barriers to competition and exchange of genetic material.

In principle, this variation could reflect some combination of physical barriers (true biogeography), short-term stochastic effects, and/or functional differentiation. Given the confounding variables of geography, time, and environmental conditions in the current collection of samples, it is difficult to definitively separate these effects, but various observations argue for functional differentiation between subtypes (i.e., they constitute distinct ecotypes). First, individual subtypes may be found in a wide range of locations; P. ubique HTCC1062 was isolated in the Pacific Ocean off the coast of Oregon [55], but closely related sequences are relatively abundant in our samples taken in the Atlantic Ocean. Second, geography per se cannot fully explain differences in subtype distributions, as multiple subtypes are found simultaneously in a single sample. Third, the collection of samples in which a given subtype was found generally exhibits similar environmental conditions. A strong independent illustration of this comes from the correlation of temperature with the distribution of *Prochlorococcus* subtypes [56]. Fourth, the extensive variation within each subtype (i.e., the fact that subtypes are not clonal populations) indicates that it cannot be chance alone that makes genetically similar organisms have similar observed distributions.

Taken together, these results argue that subtype classification is more informative for categorizing microbial populations than classification using 16S-based ribotypes, or fingerprinting techniques based on length polymorphism, such as T-RFLPs [57] or ARISA [58]. For example, the grouping of such disparate microbial populations under the umbrella P. marinus dilutes the significance of the term “species.” Indeed, numerous papers have been devoted to comparing and contrasting the differences and variability in P. marinus isolates to better understand how this particularly abundant group of organisms has evolved and adapted within the dynamic marine environment [28,52,56,59–66]. Prior to the widespread use of marker-based phylogenetic approaches, microbial systematics relied on a wide range of variables to distinguish microbial populations [67]. Subtypes bring us back to these more comprehensive approaches since they reflect the influences of a wide range of factors in the context of an entire genome.

Although subtypes are a salient feature of our data, variation within a ribotype does not stop at the level of subtypes. Variation within subtypes is so extensive that few GOS reads can be aligned at 100% identity to any other GOS read, despite the deep coverage of several taxonomic groups. Related findings have been shown for the ITS region in various organisms [2,51,52], and in a limited number of organisms for individual protein coding and intergenic regions [2,53,68]. High levels of diversity within the ribotype can be convincingly demonstrated in the 16S gene itself [69]. The applicability of these results over the entire genome were recently shown for P. marinus [28] using data from the Sargasso Sea samples taken as a pilot project for the expedition reported here [19]. We have definitively demonstrated the generality of these findings, greatly increased our understanding of the minimum number of variants of a given organism, and shown that these observations apply to the entire genome for a wide range of abundant taxonomic groups and across a wide range of geographic locations.

Average pairwise differences of several percent between overlapping P. marinus or SAR11 reads imply that this variation did not arise recently. If one uses substitution rates estimated for E. coli [70], one could conclude that on average any two P. marinus cells must have diverged millions of years ago. Mutational rates are notoriously variable and hard to estimate, and assumptions of molecular clocks are equally chancy, but clearly within-subtype variants have persisted side by side for quite some time. This raises a question related to the classic “Paradox of the Plankton”: how can so many similar organisms have coexisted for so long [71,72]? One explanation, which we favor, is that not only subtypes but also individual variants are sufficiently different phenotypically to prevent any one strain from completely replacing all others (discussed further below; see [71] for a recent theoretical treatment). An alternative is that recombination might prevent selective sweeps within ecotypes, as proposed by Cohan (reviewed in [73]).

### The Significance of Within-Subtype Variation

Given the apparent generality of subtypes and intra-subtype variation, it is important to understand if and how these subpopulations are functionally distinct. At the level of DNA sequence, a substantial fraction of substitutions are silent in terms of amino acid sequence, and others may be nonsynonymous but functionally neutral. However, two organisms that differ by 5% in their genetic sequence (e.g., 100,000 substitutions in 2 Mbp of shared sequence) will inevitably have at least minor functional differences such as in the optimal temperature or pH for the activity of some enzyme. At the level of gene content, the observation of hypervariable segments ([28] and here) implies that there is an additional dimension to functional variability. Hypervariable genomic islands with preferential insertion sites could potentially be associated with a wide range of functions, though to date they have been most closely examined for their role in pathogenicity (for a review, see [74]). However, given their apparent variability within even a single sampling site, it seems unlikely that these elements reflect a specific adaptive advantage to the local population. Identifying the source(s), diversity, and range of functionality associated with these islands by fully sequencing a large number of these segments and understanding how their individual abundances fluctuate should be quite informative.

Some might still argue that these differences must be moot for the purpose of understanding the role these organisms play in an ecosystem. Yet even small differences in optimal conditions may have profound effects. They may prevent any single genotype from being universally fittest, allowing and/or necessitating the coexistence of multiple variants [2,51,69]. Moreover, variation within subtype might afford a form of functional “buffering,” such that the population as a whole may be more stable in its ecosystem role than any one clone could be (see also [51]). That is, while any one strain of *Prochlorococcu*s might thrive and provide energy input to the rest of the community at a limited range of temperatures, light conditions, etc., the ensemble might provide such inputs over a wider range of environmental conditions. In this way, microdiversity might provide system stability or robustness through functional redundancy and the “insurance effect” (reviewed in [75]). Thus, while the extent of microdiversity suggests that knowing the behavior of any one isolate in exquisite detail might not be as useful to reductionist modeling as one might hope, this buffering could afford a more stable ensemble behavior, facilitating the development and maintenance of an ecosystem and allowing for system-level modeling.

A direct equation of subtypes with ecotypes is tempting, but not entirely clear-cut. The correlation of *PstS* distribution with phosphate abundance suggests a functional adaptation, but within *Prochlorococcus* and SAR11 the presence or absence of *PstS* subdivides subtypes without apparent respect for phylogenetic structure. This contrasts markedly with the distribution of proteorhodopsin-tuning variants within SAR11, which, despite a few convergent substitutions, are strongly congruent with phylogeny. It is interesting to ask what distinguishes pressures or adaptations that respect (or that lead to) lineage splits from those that show little or no phylogenetic structuring. These two specific examples plausibly reflect two different mechanisms (i.e., convergent but independent mutation in proteorhodopsin genes and the acquisition by horizontal transfer of genes involved in phosphate uptake). Yet, we must wonder: given the evidence that proteorhodopsin has been transferred laterally [48], and that only a small number of mutations, in some circumstances even a single base-pair change, are required to switch between the blue-absorbing and green-absorbing forms [39,40], why should proteorhodopsin variants show any lineage restriction? Perhaps this relates to the modularity of the system in question: proteorhodopsin tuning may be part of a larger collection of synergistic adaptations that are collectively not easily evolved, acquired, or lost, while the *PstS* and surrounding genes may represent a functional unit that can be readily added and removed over relatively short evolutionary time scales. If so, perhaps subtypes are indeed ecotypes, but rapidly evolving characters can lead to phenotypes that crosscut or subdivide ecotypes.

Phage provide one possible mechanism for rapid evolution of microbial populations or strains, and have been found in abundance with this and other marine metagenomic datasets [18,20]. It has been proposed that hypervariable islands are phage mediated [28]. However, there are reasons to be cautious about invoking phage as an explanation for rapidly evolving characteristics. While we see variability of *PstS* and neighboring genes in both SAR11 and P. marinus populations, this variation does not seem to be linked to recent phage activity. Initially, the distribution of *PstS* seems similar to the variation associated with the hypervariable islands, which may be phage mediated [28]. Indeed, phosphate-regulating genes including *PstS* have been identified in phage genomes [64], presumably because enhanced phosphate acquisition is required during the replication portion of their life cycle. However, the regions containing the *PstS* genes in both SAR11 and *Prochlorococcus* do not behave in the same fashion as clearly hypervariable regions, being effectively bimorphic (modulo the level of sequence variation observed elsewhere in the genome), whereas clearly hypervariable regions are so diverse that nearly every sampled clone falling in such a region appears completely unrelated to every other. Nor do the other genes in *PstS*-containing regions appear to be phage associated. These observations suggest that differences in *PstS* presence or absence arose in the distant past, or that different mechanisms are at work. It seems likely that phage may mediate lateral transfer of *PstS* and other phosphate acquisition genes, but it is unclear whether these genes then can become fixed within the population. Phage require enhanced phosphate acquisition as part of their life cycle [64], so regulatory or functional differences in these genes may limit their suitability for being acquired by the host cell for its own purposes. The rate of phage-mediated horizontal transfer of genes may reflect a combination of the gene's value to the host and to the agent mediating the transfer (e.g., phage), suggesting that *PstS* may have much greater immediate value than do proteorhodopsin genes and their variants.

In practical terms, these results highlight the limitations associated with marker-based analysis and the use of these approaches to infer the physiology of a particular microbial population. At the resolution used here, marker-based approaches are not always informative regarding differences in gene content (e.g., the *PstS* gene as well as neighboring genes), especially those associated with hypervariable segments. Though phosphate acquisition is known to vary within different strains of P. marinus [64,76], our results clearly show that this variability can happen within a single subtype (as represented by MIT9312), effectively identifying distinct ecotypes. Given the correct samples from the appropriate environments, other core genes might also show similar variation and allow us to more fully assess the reliability of reference genomes as indicators of physiological potential.

### Tools and Techniques

Analysis of the GOS dataset has benefited from the development of new tools and techniques. Many of these approaches rely on fairly well-known techniques but have been modified to take greater advantage of the metadata.

The technique of fragment recruit and the corresponding fragment recruitment plots have proven highly useful for examining the biogeography and genomic variation of abundant marine microbes when a close reference genome exists. Ultimately, this approach derives from the percent identity plots of PipMaker [27]. Similar approaches have been used to examine variation with respect to metagenomic datasets. For example, hypervariable segments and sequence variation have been visualized in P. marinus MIT9312 using the Sargasso Sea data [28] and in human gut microbes Bifidobacterium longum and Methanobrevibacter smithii [77].

Our primary advance associated with fragment recruitment plots is the incorporation of metadata associated with the isolation or production of the sequencing data. While simple in nature, the resulting plots can be extremely informative due to the volume of data being presented. Being able to present the sequence similarity and metadata visually allows a researcher to quickly identify interesting portions of the data for further examination. This is one of the first tools to make extensive use of the metadata collected during a metagenomic sequencing project. The use of sample and recruitment metadata is just the beginning. It is not difficult to imagine displaying other variations such as water temperature, salinity, phosphate abundance, and time of year with this approach. Even sample independent metadata such as phylogenetic information may produce informative views of the data. The usefulness of this and related approaches will only grow as the robust collection of metadata becomes routine and the variables that are most relevant to microbial communities are further elucidated.

The greatest limitation of fragment recruitment is the lack of appropriate reference sequences, particularly finished genomes. Using a series of modifications to the Celera Assembler referred to as “extreme assembly,” we have produced large assemblies for cultivated and uncultivated marine microbes. On its own, the extreme assembly approach would be excessively prone to producing chimeric sequences. However, when extreme assemblies are used as references for fragment recruitment, the metadata provides additional criteria to validate the sampling consistency along the length of the scaffold. Chimeric joins can be rapidly detected and avoided. This argues that future metagenomic assemblers could be specifically designed to make use of the metadata to produce more accurate assemblies, and that metagenomic assemblies will be improved by using data from multiple sources. Finding ways to represent the full diversity in these assemblies remains a pressing issue.

Extreme assembly can produce much larger assemblies but it is still limited by overall coverage. While many ribotypes are presumably present in sufficient quantities that reasonable assemblies of these genomes might be expected, this did not occur even for the most abundant organisms, including SAR11 and *P. marinus.* Many of the problems can be attributed to the diversity associated with the hypervariable segments where the effective coverage drops precipitously. If these are indeed commonplace in the microbial world, it is unlikely that complete genomes will be produced using the small insert libraries presented here. However, the ability to bin the larger sequences based on their coverage profiles across multiple samples, oligonucleotide frequency profiles, and phylogenetic markers suggests that large portions of a microbial genome can be reconstructed from the environmental data. This in turn should provide critical insights into the physiology and biochemistry of these microbial lineages that will inform culture techniques to allow cultivation of these recalcitrant organisms under laboratory conditions.

Not every technique described herein relies on metadata. The marker-less, overlap-based metagenomic comparison provides a quantitative approach to comparing the overall genetic similarity of two samples (Figures 10 and 11). In essence, genomic similarity acts as a proxy for community similarity. Marker-based approaches such as ARISA including the use of 16S sequences described herein can also be used to infer community similarity, though these approaches more aptly generate a census of the community members [51,69,78,79]. This census is biased to the extent that 16S genes can vary in copy number and relies on linkage of the marker gene to infer genome composition. While our metagenome comparison does not directly provide a census, the sensitivity can be tuned by restricting the identity of matches. This means that even subtype-level differences can be detected across samples. It would also identify the substantial gene content differences between the K12 and O157:H7 E. coli strains [12]. Such large-scale gene content differences have yet to be seen between closely related marine microbes, but may be a factor in other environments. Although the requisite amount of data will vary with the complexity of the environment or the degree of resolution required, we have found that 10,000 sequencing reads is sufficient to reliably measure the similarity of two surface water samples (unpublished data). This analysis may become a general tool for allocating sequencing resources by allowing a shallow survey of many samples followed by deep sequencing of a select number of “interesting” ones.

The application of this technique for comparing samples along with detailed analysis of fragments recruiting to a given reference sequence can also help explicate differences among communities in gene content or sequence variation. For example, recent metagenomic studies have reported differences in abundance of various gene families or differing functional roles between samples. Some of these differences correspond to plausible differences in physiology and biochemistry, such as the relative overabundance of photosynthetic or light-responsive genes in surface water samples [20,32]. Other differences however are less obvious, such as the abundance of ribosomal proteins at 130 m or the abundance of tranposase at 4,000 m [20]; some of these may reflect “taxonomic hitchhiking,” such that a sample rich in Archaea or *Firmicutes* or *Cyanobacteria,* etc., has an overrepresentation of genes more reflective of their recent evolutionary history than of a response to environmental conditions. Being able to control or account for these taxonomic effects is crucial to understanding how microbial populations have adapted to environmental conditions and how they may behave under changing conditions. The metagenomic comparison method described here provides a new tool to more accurately measure the impact of taxonomic effects.

In conclusion, this study reveals the wealth of biological information that is contained within large multi-sample environmental datasets. We have begun to quantify the amount and structure of the variation in natural microbial populations, while providing some information about how these factors are structured along phylogenetic and environmental factors. At the same time, many questions remain unanswered. For example, although microbial populations are structured and therefore genetically isolated, we do not understand the mechanisms that lead to this isolation. Their isolation seems contradictory given overwhelming evidence that horizontal gene transfer associated with hypervariable islands is a common phenomenon in marine microbial populations. Whatever the mechanism, the role and rate at which gene exchange occurs between populations will be crucial to understanding population structure within microbial communities and whether these communities are chance associations or necessary collections. The hypervariable islands could be a source for tremendous genetic innovation and novelty as evidenced by the rate of discovery of novel protein families in the GOS dataset [18]. However, it is not clear whether these entities are the main source of this novelty or whether this novelty resides in the vast numbers of rare microbes [4] that cannot be practically accessed using current metagenomic approaches. Altogether, this research reaffirms our growing wealth and complexity of data and paucity of understanding regarding the biological systems of the oceans.

## Materials and Methods

### Sampling sites.

A more detailed description of the sampling sites provides additional context in which to understand the individual samples. The northernmost site (GS05) was at Compass Buoy in the highly eutrophic Bedford Basin, a marine embayment encircled by Halifax, Nova Scotia, that has a 15-y weekly record of biological, physical, and chemical monitoring (http://www.mar.dfo-mpo.gc.ca/science/ocean/BedfordBasin/index.htm). Other temperate sites included a coastal station sample near Nova Scotia (GS4), a station in the Bay of Fundy estuary at outgoing tide (GS06), and three Gulf of Maine stations (GS02, GS03, and GS07). These were followed by sampling coastal stations from the New England shelf region of the Middle Atlantic Bight (Newport Harbor through Delaware Bay; GS08–GS11). The Delaware Bay (GS11) was one of several estuary samples along the Global Expedition path. Estuaries are complex hydrodynamic environments that exhibit strong gradients in oxygen, nutrients, organic matter, and salinity and are heavily impacted by anthropogenic nutrients. The Chesapeake Bay (GS12) is the largest estuary in the United States and has microbial assemblages that are diverse mixtures of freshwater and marine-specific organisms [80]. GS13 was collected near Cape Hatteras, North Carolina, inside and north of the Gulf Stream, and GS14 was taken along the western boundary frontal waters of the Gulf Stream off the coast of Charleston, South Carolina. The vessel stopped at five additional stations as it transited through the Caribbean Sea (GS15–GS19) to the Panama Canal. In Panama, we sampled the freshwater Lake Gatun, which drains into the Panama Canal (GS20). The first of the eastern Pacific coastal stations GS21, GS22, and GS23 were sampled on the way to Cocos Island (~500 km southwest of Costa Rica), followed by a coastal Cocos Island sample (GS25). Near the island, ocean currents diverge and nutrient rich upwellings mix with warm surface waters to support a highly productive ecosystem. Cocos Island is distinctive in the eastern Pacific because it belongs to one of the first shallow undersea ridges in the region encountered by the easterly flowing North Equatorial Counter/Cross Current in the Far Eastern Pacific [81,82]. After departing Cocos Island, the vessel continued southwest to the Galapagos Islands, stopping for an open ocean station (GS26). An intensive sampling program was then conducted in the Galapagos. The Galapagos Archipelago straddles the equator 960 km west of mainland Ecuador in the eastern Pacific. These islands are in a hydrographically complex region due to their proximity to the Equatorial Front and other major oceanic currents and regional front systems [83]. The coastal and marine parts of the Galapagos Islands ecosystem harbor an array of distinctive habitats, processes, and endemic species. Several distinct zones were targeted including a shallow-water, warm seep (GS30), below the thermocline in an upwelling zone (GS31), a coastal mangrove (GS32), and a hypersaline lagoon (GS33). The last stations were collected from open ocean sites (GS37 and GS47) and a coral reef atoll lagoon (GS51) in the immense South Pacific Gyre. The open ocean samples come from a region of lower nutrient concentrations where picoplankton are thought to represent the single most abundant and important factor for biogeochemical structuring and nutrient cycling [84–87]. In the atoll systems, ambient nutrients are higher, and bacteria are thought to constitute a large biomass that is one to three times as large as that of the phytoplankton [88–90].

### Sample collection.

A YSI (model 6600) multiparameter instrument (http://www.ysi.com) was deployed to determine physical characteristics of the water column, including salinity, temperature, pH, dissolved oxygen, and depth. Using sterilized equipment [91], 40–200 l of seawater, depending on the turbidity of the water, was pumped through a 20-μm nytex prefilter into a 250-l carboy. From this sample, two 20-ml subsamples were collected in acid-washed polyethylene bottles and frozen (−20 °C) for nutrient and particle analysis. At each station the biological material was size fractionated into individual “samples” by serial filtration through 20-μm, 3-μm, 0.8-μm, and 0.1-μm filters that were then sealed and stored at −20 °C until transport back to the laboratory. Between 44,160 and 418,176 clones per station were picked and end sequenced from short-insert (1.0–2.2 kb) sequencing libraries made from DNA extracted from filters [19]. Data from these six *Sorcerer II* expedition legs (37 stations) were combined with the results from samples in the Sargasso Sea pilot study (four stations; GS00a–GS00d and GS01a–GS01c; [19]. The majority of the sequence data presented came from the 0.8- to 0.1-μm size fraction sample that concentrated mostly bacterial and archaeal microbial populations. Two samples (GS01a, GS01b) from the Sargasso Sea pilot study dataset and one GOS sample (GS25) came from other filter size fractions (Table 1).

### Filtration and storage.

Microbes were size fractionated by serial filtration through 3.0-μm, 0.8-μm, and 0.1-μm membrane filters (Supor membrane disc filter; Pall Life Sciences, http://www.pall.com), and finally through a Pellicon tangential flow filtration (Millipore, http://www.millipore.com) fitted with a Biomax-50 (polyethersulfone) cassette filter (50 kDa pore size) to concentrate a viral fraction to 100 ml. Filters were vacuum sealed with 5 ml sucrose lysis buffer (20 mM EDTA, 400 mM NaCl, 0.75 M sucrose, 50 mM Tris-HCl [pH 8.0]) and frozen to −20 °C on the vessel until shipment back to the Venter Institute, where they were transferred to a −80 °C freezer until DNA extraction. Glycerol was added (10% final concentration) as a cryoprotectant for the viral/phage sample.

### DNA isolation.

In the laboratory, the impact filters were aseptically cut into quarters for DNA extraction. Unused quarters of the filter were refrozen at −80 °C for storage. Quarters used for extraction were aseptically cut into small pieces and placed in individual 50-ml conical tubes. TE buffer (pH 8) containing 50 mM EGTA and 50 mM EDTA was added until filter pieces were barely covered. Lysozyme was added to a final concentration of 2.5 mg/ml^−1^, and the tubes were incubated at 37 °C for 1 h in a shaking water bath. Proteinase K was added to a final concentration of 200 μg/ml^−1^, and the samples were frozen in dry ice/ethanol followed by thawing at 55 °C. This freeze–thaw cycle was repeated once. SDS (final concentration of 1%) and an additional 200 μg/ml^−1^ of proteinase K were added to the sample, and samples were incubated at 55 °C for 2 h with gentle agitation followed by three aqueous phenol extractions and one phenol/chloroform extraction. The supernatant was then precipitated with two volumes of 100% ethanol, and the DNA pellet was washed with 70% ethanol. Finally, the DNA was treated with CTAB to remove enzyme inhibitors. Size fraction samples not utilized in this study were archived for future analysis.

### Library construction.

DNA was randomly sheared via nebulization, end-polished with consecutive BAL31 nuclease and T4 DNA polymerase treatments, and size-selected using gel electrophoresis on 1% low-melting-point agarose. After ligation to BstXI adapters, DNA was purified by three rounds of gel electrophoresis to remove excess adapters, and the fragments were inserted into BstXI-linearized medium-copy pBR322 plasmid vectors. The resulting library was electroporated into *E. coli.* To ensure construction of high-quality random plasmid libraries with few to no clones with no inserts, and no clones with chimeric inserts, we used a series of vectors (pHOS) containing BstXI cloning sites that include several features: (1) the sequencing primer sites immediately flank the BstXI cloning site to avoid excessive resequencing of vector DNA; (2) elimination of strong promoters oriented toward the cloning site; and (3) the use of BstXI sites for cloning facilitates the preparation of libraries with a low incidence of no-insert clones and a high frequency of single inserts. Clones were sequenced from both ends to produce pairs of linked sequences representing ~820 bp at the end of each insert.

### Template preparation.

Libraries were transformed, and cells were plated onto large format (16 × 16cm) diffusion plates prepared by layering 150 ml of fresh molten, antibiotic-free agar onto a previously set 50-ml layer of agar containing antibiotic. Colonies were picked for template preparation using the Qbot or QPix colony-picking robots (Genetix, http://www.genetix.com), inoculated into 384-well blocks containing liquid media, and incubated overnight with shaking. High-purity plasmid DNA was prepared using the DNA purification robotic workstation custom-built by Thermo CRS (http://www.thermo.com) and based on the alkaline lysis miniprep [92]. Bacterial cells were lysed, cell debris was removed by centrifugation, and plasmid DNA was recovered from the cleared lysate by isopropanol precipitation. DNA precipitate was washed with 70% ethanol, dried, and resuspended in 10 mM Tris HCl buffer containing a trace of blue dextran. The typical yield of plasmid DNA from this method is approximately 600–800 ng per clone, providing sufficient DNA for at least four sequencing reactions per template.

### Automated cycle sequencing.

Sequencing protocols were based on the di-deoxy sequencing method [93]. Two 384-well cycle-sequencing reaction plates were prepared from each plate of plasmid template DNA for opposite-end, paired-sequence reads. Sequencing reactions were completed using the Big Dye Terminator chemistry and standard M13 forward and reverse primers. Reaction mixtures, thermal cycling profiles, and electrophoresis conditions were optimized to reduce the volume of the Big Dye Terminator mix (Applied Biosystems, http://www.appliedbiosystems.com) and to extend read lengths on the AB3730xl sequencers (Applied Biosystems). Sequencing reactions were set up by the Biomek FX (Beckman Coulter, http://www.beckmancoulter.com) pipetting workstations. Robots were used to aliquot and combine templates with reaction mixes consisting of deoxy- and fluorescently labeled dideoxynucleotides, DNA polymerase, sequencing primers, and reaction buffer in a 5 μl volume. Bar-coding and tracking promoted error-free template and reaction mix transfer. After 30–40 consecutive cycles of amplification, reaction products were precipitated by isopropanol, dried at room temperature, and resuspended in water and transferred to one of the AB3730xl DNA analyzers. Set-up times were less than 1 h, and 12 runs per day were completed with average trimmed sequence read length of 822 bp.

### Fosmid end sequencing.

Fosmid libraries [24] were constructed using approximately 1 μg DNA that was sheared using bead beating to generate cuts in the DNA. The staggered ends or nicks were repaired by filling with dNTPs. A size selection process followed on a pulse field electrophoresis system with lambda ladder to select for 39–40 Kb fragments. The DNA was then recovered from a gel, ligated to the blunt-ended pCC1FOS vector, packaged into lambda packaging extracts, incubated with the host cells, and plated to select for the clones containing an insert. Sequencing was performed as described for plasmid ends.

### Metagenomic assembly.

Assembly was conducted with the Celera Assembler [21], with modifications as follows. The “genome length” was artificially set at the length of the dataset divided by 50 to allow unitigs of abundant organisms to be treated as unique, as previously described [19]. Several distinct assemblies were computed. In the primary assembly, all pairs of mated reads were tested to see whether the paired reads overlapped one another; if so, they were merged into a single pseudo-read that replaced the two original reads; further, only overlaps of 98% identity or higher were used to construct unitigs. A second assembly was conducted in the same fashion with the exception of using a 94% identity cutoff to construct unitigs. Finally, series of assemblies at various stringencies were computed for subsets of the GOS data; in these assemblies, overlapping mates were not preassembled and the Celera Assembler code was modified slightly to allow for overlapping and multiple sequence alignment at lower stringency.

### Construction of a low-identity overlap database.

An all-against-all comparison of unassembled (but merged and duplicate-stripped) sequences from the combined dataset was performed using a modified version of the overlapper component of the Celera Assembler [21]. The code was modified to find overlap alignments (global alignments allowing free end gaps) starting from pairs of reads that share an identical substring of at least 14 bp. An alignment extension was then performed with match/mismatch scores set to yield a positive outcome if an overlap alignment was found with ≥65% identity. Overlaps involving alignments of ≥40 bp were retained for various analyses. For the GOS dataset described here, this process resulted in a dataset of 1.2 billion overlaps. Due to the 14-bp requirement and certain heuristics for early termination of apparently hopeless extensions, not all alignments at ≥65% were found. In addition, some of the lowest-identity overlaps are bound to be chance matches; however, this was a relatively uncommon event. Approximately one in 5 × 10^6^ pairs of 800-bp random sequences (all sites independent, A = C = G = T = 25%) can be aligned to overlap ≥40 bp at ≥65% identity using the same procedure. At a 70% cutoff, the value is reduced to one in 4 × 10^7^, and one in 5 × 10^8^ at a 75% cut off.

### Extreme assembly.

Like many assembly algorithms, the extreme assembler proceeds in three phases: overlap, layout, and consensus. The overlap phase is provided by the all-against-all comparison described above. The consensus phase is performed by a version of the Celera Assembler, modified to accept higher rates of mismatch. The layout phase begins with a single sequencing read (“seed”) that is chosen at random or specified by the user and is considered the “current” read. The following steps are performed off one or both ends of the seed. (1) Starting from the current fragment end, add the fragment with the best overlap off that end and mark the current fragment as “used,” thus making the added fragment the new current fragment. (2) Mark as used any alternative overlap that would have resulted in a shorter extension. The simplest notion of “best overlap” is simply the one having the highest identity alignment, but more complicated criteria have certain advantages. A simple but useful refinement is to favor fragments whose other ends have overlaps over those which are dead ends. For an unsupervised extreme assembly, when the sequence extension terminates because there are no more overlaps, a new unused fragment is chosen as the next seed and the process is repeated until all fragments have been marked used.

### Construction of multiple SAR11 variants.

Sequencing reads mated to SAR11-like 16S sequences but themselves outside of the ribosomal operon (*n* = 348) were used as seeds in independent extreme assemblies. Since the assemblies were independent, the results were highly redundant, with a given chain of overlapping fragments typically being used in multiple assemblies. A subset of 24 assemblies that shared no fragments over their first 20 kb was identified as follows. (1) Connected components were determined in a graph defined by nodes corresponding to extreme assemblies. If the assemblies shared at least one fragment in the first 20 kb of each assembly, the two nodes were connected by an edge. (2) A single assembly was chosen at random from each of the connected components. The consensus sequence over the 20-kb segment of each such representative was used as the reference for fragment recruitment.

### Phylogeny.

Phylogenies of sequences homologous to a given portion of a reference sequence (typically 500 bp) were determined in the following manner. A set of homologous fragments was identified based on fragment recruitment to the reference as described above. Fragments that fully spanned the segment of interest and had almost full-length alignments to the reference sequence of a user-defined percent identity (typically, 70%) were used for further analysis. A preliminary master–slave multiple sequence alignment of the recruited reads (slaves) to the reference segment (master) was performed with a modified version of the consensus module of the Celera Assembler. Based on this alignment, reads were trimmed to the portion aligning to the reference segment of interest. A refined multiple sequence alignment was then computed with MUSCLE [94]. Distance based phylogenies were computed using the programs DNADIST and NEIGHBOR from the PHYLIP package [95] using default settings. Trees were visualized using HYPERTREE [96].

### Measurement of library-to-library similarity.

Based on the low-identity overlap database described above, the similarity of a library *i* to another library *j* at a given percent identity cutoff was computed as follows. For each sequence *s* of *i*, let *n_s,i_* = the number of overlaps to other fragments of *i* satisfying the cutoff; *n_s,j_* = the number of overlaps to fragments of *j* satisfying the cutoff; and *f_s,i_* = *n_s,i_*/(*n_s,i_* + *n_s,j_*) *=* fraction of reads overlapping *s* from *i* or *j* that are from *i*.

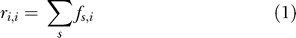


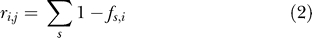


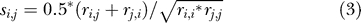


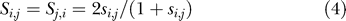



A read that can be overlapped to another at sufficiently high-sequence identity was taken to indicate that they were from similar organisms, and, relatedly, that similar genes were present in the samples. Only reads with such overlaps contributed to the calculation. Other reads reflect genes or segments of genomes that were so lightly sampled (i.e., at such low abundance) that they were not informative regarding the similarity of two samples. Consequently, the analysis automatically corrects for differences in the amount of sequencing, and can be computed over sets of samples that vary considerably in diversity. The resulting measure of similarity *S_i,j_* takes on a value between 0 and 1, where 0 implies no overlaps between *i* and *j*, and 1 implies that a fragment from *i* and a fragment from *j* are as likely to overlap one another as are two fragments from *i* or two fragments from *j*. As with the Bray-Curtis coefficient [97], abundance of categories affects the computation. In an idealized situation where two libraries can each be divided into some number *k* of “species” at equal abundance, and the libraries have *l* of the species in common, the similarity statistic will approach *l*/*k* for large samplings*;* in this sense, *S_i,j_ = x* indicates that the two samples share approximately a fraction *x* of their genetic material. It is frequently useful to define *D_i,j_ =* 1 − *S_i,j_*, the “dissimilarity” or distance between two samples.

### Ribotype clustering and identification of representatives.

An all-against-all comparison of predicted 16S sequences was performed to determine the alignment between pairs of overlapping sequences using a version of an extremely fast bit-vector algorithm [98]. A hierarchical clustering was determined using percent-mismatch in the resulting alignments as the distance between pairs of sequences. Order of clustering and cluster identity scores were based on the average-linkage criterion, with distances between nonoverlapping partial sequences treated as missing data. Ribotypes were the maximal clusters with an identity score above the cutoff (typically 97%). Representative sequences were chosen for each cluster based on both length and highest average identity to other sequences in the cluster.

### Taxonomic classification.

Taxonomic classification of 16S sequences was conducted using phylogenetic techniques based on clade membership of similar sequences with 16S sequences with defined taxonomic membership. Representative sequences from clustered sequences were analyzed as described previously [19,99] and by addition into an ARB database of small subunit rDNAs [100,101]. Results were spot-checked against the Ribosomal Database Project II Classifier server [102] and the taxonomic labels of the best BLASTN hits against the nonredundant database at NCBI.

### Fragment recruitment.

Global ocean sequences were aligned to genomic sequences of different bacteria and phage using NCBI BLASTN [26]. The following blast parameters were designed to identify alignments as low as 55% identity that could contain large gaps: -F “m L” -U T -p blastn -e 1e-4 -r 8 -q -9 -z 3000000000 -X 150. Reads were filtered in several steps to identify the reads that were aligned over more or less their entire length. Reads had to be aligned for more than 300 bp at >30% identity with less than 25 bp of unaligned bases on either end, or reads had to be aligned over more than 100 bp at >30% identity with less than 20 bp of overhang off either end. Identity was calculated ignoring gaps. In some instances a read might be placed, but the mate would not be placed under these criteria. In such cases, if 80% or more of the mate were successfully aligned, then the mate would be rescued and considered successfully aligned.

### Generation of shredded artificial reads from finished genomes.

Random pieces of DNA from the genome in question with a length between 1,800 to 2,500 bp were selected. For each piece a read length N1 was selected from the distribution of lengths using the GOS dataset. If that GOS sequence had a mate pair, then a second length N2 was again randomly selected. The length N1 was used to generate a read from the 5′ end of the DNA. The piece of DNA was then reverse complemented and if appropriate, a second length N2 was used to generate a second read. The relationship between these two reads was then recorded and used to produce a fasta file. This approach successfully mimics the types of reads found in the GOS data with similar rates of missing mates.

### Abundance of proteorhodopsin variants.

A total of 2,644 proteorhodopsin genes were identified from the clustered open reading frames derived from the GOS assembly [18]. These genes could be linked back to 3,608 GOS clones. Open reading frames were predicted from these clones as described in [18]. The peptide sequences were aligned with NCBI blastpgp with the following parameters: -j 5 -U T -e 10 -W 2 -v 5 -b 5000 -F “m L” -m 3. The search was performed with a previously described blue-absorbing proteorhodopsin protein BPR (gi|32699602) as the query. The amino acid associated with light absorption is found within a short conserved motif RYVDWLLTVPL*IVEF, where the asterisk indicates tuning amino acid [39–41]. In total, 1,938 clones were found to contain this motif. Clones and the sample metadata were then associated with the tuning amino acid to determine the relative abundance of the different amino acids at these positions. Clones could be associated with SAR11 if both mated sequencing reads (when available) were recruited to P. ubique HTCC1062.

### Site abundance estimates and comparisons.

Given a set of genes identified on the GOS sequences, we can identify the scaffolds on which these genes were annotated. A vector indicating the number of sequences contributed by every sample is determined for every gene. This vector reflects the number of sequences from every sample that assembled into the scaffold on which the gene was identified after normalizing for the proportion of scaffold covered by the gene. For example, if a 10-kb scaffold contains a 1-kb gene, then each sample will contribute one GOS sequence for every ten GOS sequences it contributed to the entire scaffold. The vectors are then summed and normalized to account for either the total number of GOS sequences obtained from each sample or based on the number of typically single copy *recA* genes (identified as in [18]). Unless stated otherwise, *recA* was used to normalize abundance across samples. When comparisons using groups of samples were performed, the average value for the samples was compared.

### Oligonucleotide composition profile.

A 1-D profile representing oligonucleotide frequencies was computed as follows. A sequence was converted into a series of overlapping 10,000-bp segments, each segment offset by 1,000 bp from the previous one, using perl and shell scripting. Dinucleotide frequencies are computed on each segment using a C program written for this purpose. Higher-order oligonucleotides were examined and gave similar results for the genomes of interest. Remaining calculations were performed using the R package [103]. Principle component analysis (function *princomp* with default settings) was applied to the matrix of frequencies per window position. The value of the first component for each position was normalized by the standard deviation of these values, and truncated to the range [−5, 5]. For visualizations, the resulting values were plotted at the center of each window.

### Estimating frequency of large-scale translocations and inversions.

The unrecruited mated sequencing reads of reads recruited to P. marinus MIT9312 at or above 80% identity were examined. An unrecruited mate indicated a potential translocation or inversion if it aligned to the MIT9312 genome in two and only two distinct alignments separated by at least 50 kb, if each aligned portion was at least 250 bp long, if there was less than 100 bp of unaligned sequence and no more than 100 bp of overlapping sequence between the two aligned portions in read coordinates, and if each aligned portion was anchored to one end of the sequencing read with less than 25 bp of unaligned sequence from each end. In total, 18 rearrangements were identified, six of which appear to be unique events.

The rate of discovery was estimated by determining the number of rearrangements in a given volume of sequence. We estimated the volume of sequence that was potentially examined by identifying recruited mated sequencing reads that fit the “good” category (i.e., which were recruited in the correct orientation at the expected distance from each other). For a given read, if the mate was recruited at greater than or equal to 80% identity, then the expected amount of sequence examined should be the current (as opposed to mate) read length minus 500 bp. This produces an estimate of the search space to be ~47 Mbp. Given 18 rearrangements, this leads to an estimate of one rearrangement per 2.6 Mbp.

### Quantification and assessment of sequences associated with gaps.

GOS reads assigned to the “missing mate” category that were recruited at greater than 80% identity outside the gap in question were identified. The mates of these reads were then identified and clustering was attempted with Phrap (http://www.phrap.org). Reads that were incorporated end to end into the Phrap assemblies were identified. For most small gaps a single assembly included all the missing mate reads and identified the precise difference between the reference and the environmental sequences. For the hypervariable segments, most of the reads failed to assemble at all, and those that did show greater sequence divergence than typically seen. In the case of SAR11-recruited reads, to increase the number of reads associated with the hypervariable gaps we identified reads that did not recruit to the P. ubique HTCC1062 but aligned in a single HSP (high-scoring pair) over at least 500 bp with one end unaligned because it extended into the hypervariable gap.

### Data and tool release.

To facilitate continued analysis of this and other metagenomic datasets, the tools presented here along with their source code will be available via the Cyberinfrastructure for Advanced Marine Microbial Ecology Research and Analysis (CAMERA) website (http://camera.calit2.net). The dataset and associated metadata will be accessible via CAMERA (using the dataset tag CAM_PUB_Rusch07a). Given the exceptional abundance of *Burkholderia* and *Shewanella* sequences in the first Sargasso Sea sample and the feeling that these may be contaminants, we are also providing a list of the scaffold IDs and sequencing read IDs associated with these organisms to facilitate analyses with or without the sequences. In addition to CAMERA, the GOS scaffolds and annotations will be available via the public sequence repositories such as NCBI (http://www.ncbi.nlm.nih.gov/entrez/query.fcgi?db=genomeprj&cmd=Retrieve&dopt=Overview&list_uids=13694), and the reads will be available via the Trace Archive (http://www.ncbi.nlm.nih.gov/Traces/trace.cgi?).

## Supporting Information

Poster S1 Fragment Recruitment of GOS Data to Finished Microbial Genomes (21 MB PDF)

### Accession Numbers

The GenBank (http://www.ncbi.nlm.nih.gov/Genbank) accession number for proteorhodopsin protein BPR is gi|32699602.

## Abbreviations

CAMERA: Cyberinfrastructure for Advanced Marine Microbial Ecology Research and Analysis
GOS: Global Ocean Sampling
NCBI: National Center for Biotechnology Information

## Note Added in Proof

Recently, Gomez-Consarnau et al. provided credible evidence for the biological role of proteorhodopsins [104]. These results indicate that proteorhodopsins blur the line between heterotrophic and autotrophic microbes by allowing a wide range of organisms to harness light energy for respiration and growth. This reinforces the notion that the differential distribution of proteorhodopsin variants identified here reflects functional adaptation to the wavelengths of available light. Furthermore, these adaptations may be driven by the makeup of the microbial community. Thus, these distributional differences could reflect competition between microbes for light resources.
