# Supplementary material for: The Sorcerer II Global Ocean Sampling Expedition: Northwest Atlantic through Eastern Tropical Pacific
Source: PLoS Biol. 2007 Mar 13;5(3):e77. doi: 10.1371/journal.pbio.0050077 (PMC1821060; doi:10.1371/journal.pbio.0050077)
Supplement: Poster S1 — (21 MB PDF) [file pbio.0050077.sd001.pdf]

J. Craig Venter™ INSTITUTE
www.venterinstitute.org

PLoS BIOLOGY
www.plosbiology.org

Fragment Recruitment of Global Ocean Sampling Data to Finished Microbial Genomes

Fragment recruitment plots are powerful tools that use environmental metagenomic data to explore variation in structure, geography, and sequence relative to a reference sequence or genome. Sequence similarity tools were used to recruit GOS sequences to the given reference sequence. Only GOS sequences that could be aligned along nearly their entire length were recruited. The position and identity where a GOS sequence recruited to a reference genome was plotted. Additional information was added to the plots by coloring individual GOS sequencing reads according to additional criteria or metadata.

While any genome, indeed any sequence, can be used as a reference, certain genomes are more informative than others. Depicted here are fragment recruitment plots to the three marine microbial genomes that recruited the greatest number of GOS sequences. *Pelagibacter ubique* HTCC1062[1] is a widely distributed highly abundant, heterotrophic alpha-proteobacteria known for its extremely small compact genome. *Synechococcus* WH8102[2] is a widely distributed, moderately abundant autotrophic cyanobacteria. *Prochlorococcus marinus* MIT9312[3] is an isolate from the most abundant clade of surface water autotrophic cyanobacteria and is related to *Synechococcus*. MIT9312 is largely restricted to nutrient poor warm tropical waters. Each of these organisms is known to be highly abundant in the world's oceans and play an important role in global carbon fixation and/or nutrient cycling.

Every GOS sequence that was successfully recruited to a reference sequence was plotted as a small colored bar at a given position (x-axis) and percent identity (y-axis). Two different color schemes are used in the plots presented here.

The **Sample Metadata** color scheme conveys information about the sampling environment and experimental conditions under which a given sequence was produced[4]. This color scheme is useful for identifying subtypes within the fragment recruitment plots.

The **Mate Pairing Metadata** is useful for identifying differences in synteny or genome structure between the reference sequence and environmental populations. This second scheme was based on the paired end shotgun sequencing strategy used to analyze the GOS samples. The paired sequencing reads are referred to as mated sequencing reads. Colors under this scheme were assigned based on the relative position along the reference and the orientation that two mated sequencing reads were recruited[5].

Each of the reference genomes is shown with the GOS sequences colored using the sample metadata. Each genome shows a distinct and fairly uniform pattern of recruitment. The GOS sequences were recruited non-randomly, instead showing considerable structure. In these examples, the GOS sequences form one or more bands that zigzag along the genome at a fairly constant identity.

The continuity of the GOS sequences that make up the zigzag indicates shared synteny between the environmental populations and the reference genome. The zigzag is particularly prominent on the *P. marinus* MIT9312 genome. Every band seems to consist of GOS sequences from a particular set of samples.

Strikingly there are large segments on all three genomes where the number of GOS sequences is greatly reduced. At a minimum, these recruitment gaps indicate that portions of the genome are rare in the environments that were sampled. Several interesting segments on each of the genomes have been blown up to highlight interesting features.

Genome composition has been measured to identify possible sites of lateral gene transfer. Tetra-nucleotide frequencies in overlapping 7.5kb intervals were compared to the overall tetra-nucleotide frequency of the entire genome. The standard deviation of the frequencies over these 7.5kb intervals from the mean is shown as a solid black line on the genomes of HTCC1062 and WH8102 or a rust colored line on MIT9312. Tetra-nucleotide frequencies vary by up to 5 standard deviations both positively and negatively from the mean, which occurs at the midline of each plot (at 75% identity). Many of the strongest deviations co-occur with recruitment gaps, possibly signifying that those segments of the genome were acquired by lateral gene transfer.

[1] Giovannoni SJ, Tripp HJ, Givan S, Podar M, Vergin KL, et al. (2005) Genome Streamlining in a Cosmopolitan Oceanic Bacterium. Science 309: 1242-1245.

[2] Palenik B, Brahamsha B, Larimer FW, Land M, Hauser L, et al. (2003) The Genome of a Motile Marine Synechococcus. Nature 424: 1037-1042.

[3] Rocap G, Larimer FW, Lamerdin J, Malfatti S, Chain P, et al. (2003) Genome divergence in two Prochlorococcus ecotypes reflects oceanic niche differentiation. Nature 424: 1042-1047.

[4] Rusch DB, Halpern AL, Sutton G, Heidelberg KB, Williamson S, et al. (2007) The *Sorcerer II* Global Ocean Sampling expedition: Northwest Atlantic through eastern tropical Pacific. PLoS Biol 5: e77. doi:10.1371/journal.pbio.0050077

[5] Rusch DB, Halpern AL, Sutton G, Venter JE, Yooseph S, et al. (submitted) Putting environmental metagenomic data in context.

A "Long" Mates Indicate a Large Insertion Event

The presence of mated GOS sequences that are spaced farther apart than expected ("long" mates) flanking a recruitment gap ("missing" mates not shown) indicates that the insertion is specific to the reference genome and not present in the environmental populations. Here, the site distribution of the flanking long mated sequences is shown. This distribution is highly skewed towards the Sargasso Sea samples suggesting that the presence of an insert at this location is sample specific. These results are consistent with the hypothesis that this segment of the genome is occupied by a hyper-variable segment in many samples but is rarely occupied in the Sargasso samples.

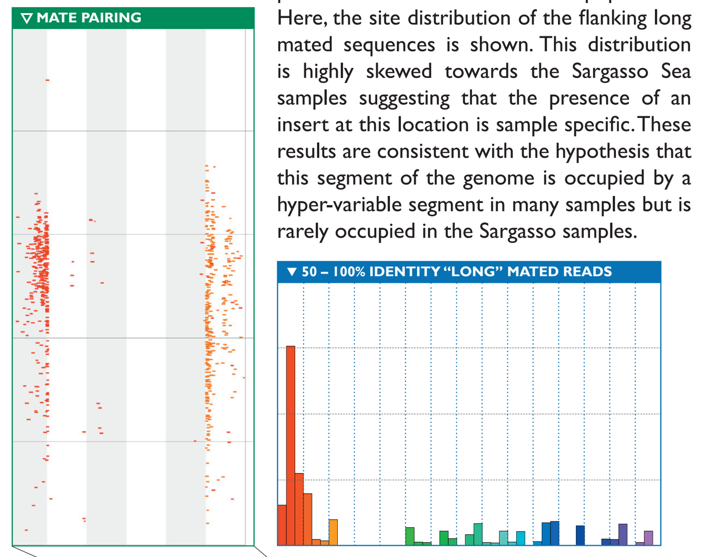

B "Missing" Mates Border Breaks in Synteny

Small recruitment gaps identify breaks in synteny. Shown here are two small deletions specific to the HTCC1062 strain. Breaks in synteny are flanked by reads labeled as "missing" mates. A missing mate is defined here as a GOS sequence whose mated sequencing read was not recruited to the HTCC1062 genome. Notice that the highest identity band of GOS sequences seems to disappear in the left half of the magnified segment. The absence of missing mates flanking the disappearance indicates this is not a break in synteny. In this instance the GOS sequences are recruited at substantially lower identity than expected.

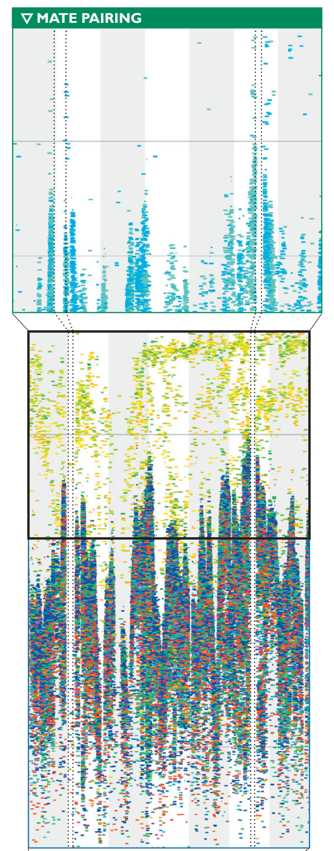

C Hyper-variable Recruitment Gaps

Recruitment gaps typically reflect breaks in synteny and therefore are flanked by "missing" mates. If this gap were due to a simple insertion specific to HTCC1062, it would also be flanked by "long" mates. If the gap contained toxic or unclonable sequences then there would not be flanking "missing" mates. A recruitment gap indicates that that segment of the genome is rare or absent in the GOS samples examined. In this case the gap identifies a hyper-variable segment that contains a different insert in nearly every clone that was examined. This gap also has a tetra-nucleotide frequency that is approximately three standard deviations from the norm suggesting this segment may be the result of lateral gene transfer.

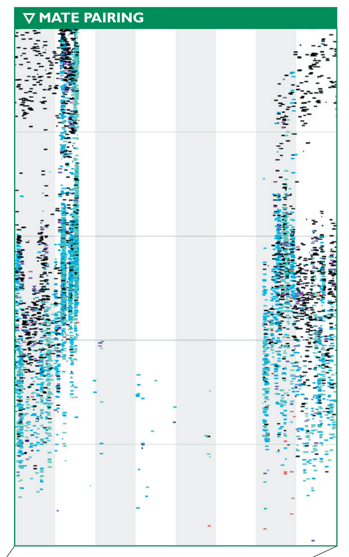

D Stratification of Recruited Fragments

Sequences from different samples are preferentially recruited at particular sequence identities. On *Pelagibacter ubique* HTCC1062 the GOS data are recruited into three distinct bands each of which consists of sequences from a wide distribution of samples. These patterns are maintained more or less uniformly along the length of the genome. Combining all three bands shows the distribution of HTCC1062-like sequences to be remarkably uniform across all the marine samples collected on 0.1 micron filters.

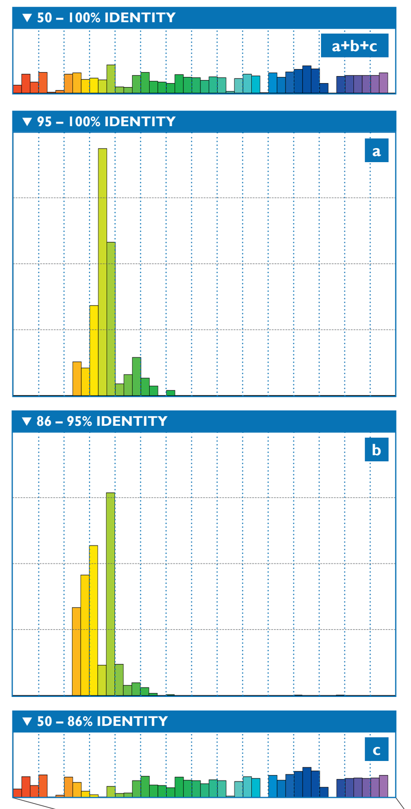

E Sample-specific Segment of *Pelagibacter ubique* HTCC1062

While many gaps are conspicuous because they fail to recruit any GOS sequences, others are subtle showing specific geographic recruitment patterns. This segment almost exclusively recruits GOS sequences from the Atlantic, Gulf of Mexico, and Caribbean samples as shown in the histogram. Many of the genes in this segment are involved in phosphate acquisition including the ortholog of a phosphate binding protein (*PstS*) gene that is found in the Atlantic-specific segment of *Prochlorococcus marinus* MIT9312.

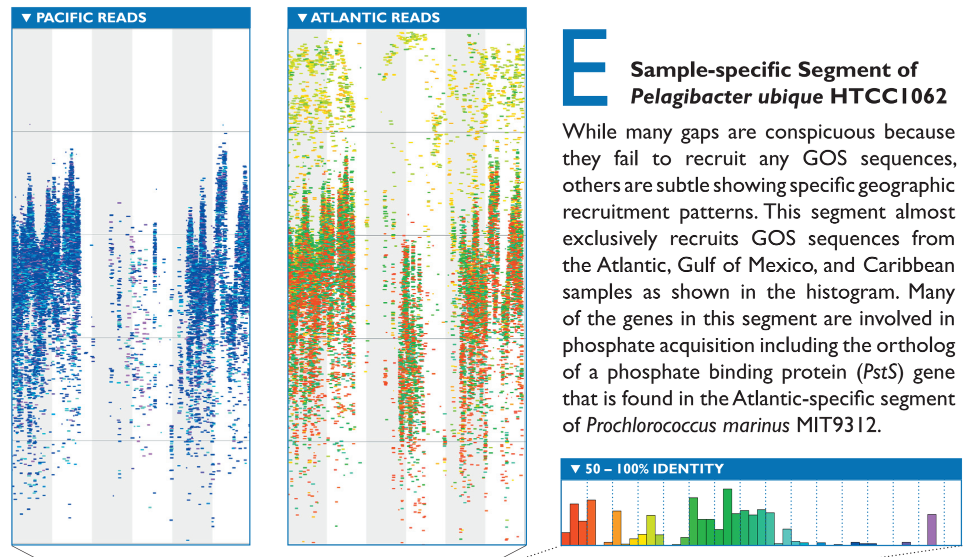

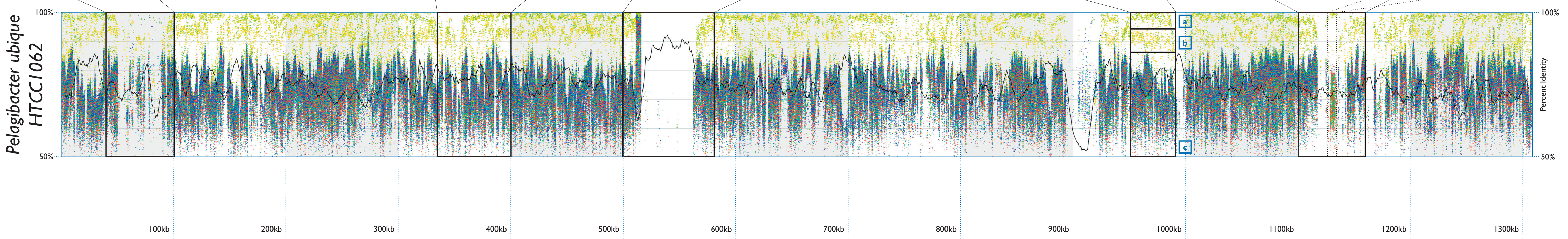

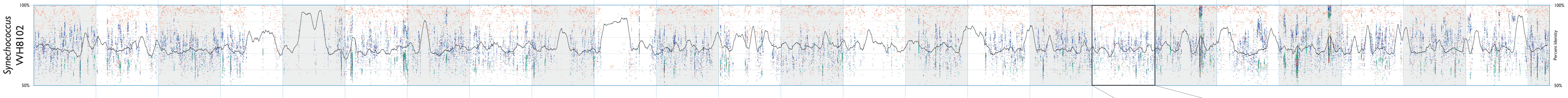

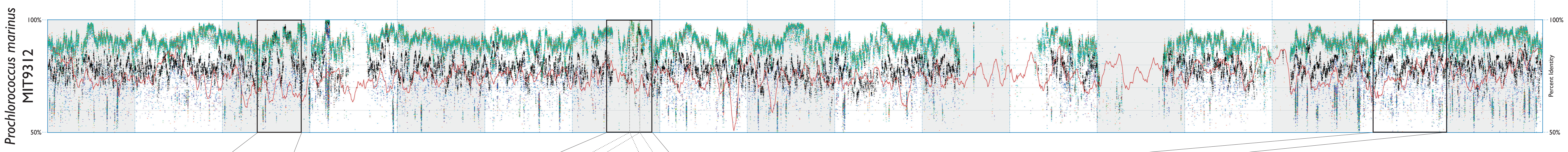

F Sample-Specificity and Stratification in *Synechococcus* WH8102

GOS sequences highly similar to WH8102 are almost exclusively from the Sargasso Sea samples. At lower identity (72-83 percent) a large number of sequences from the Pacific show similarity to this strain of *Synechococcus*. *Synechococcus*-like sequences are highly abundant in the North Seamore Island sample (station GS34).

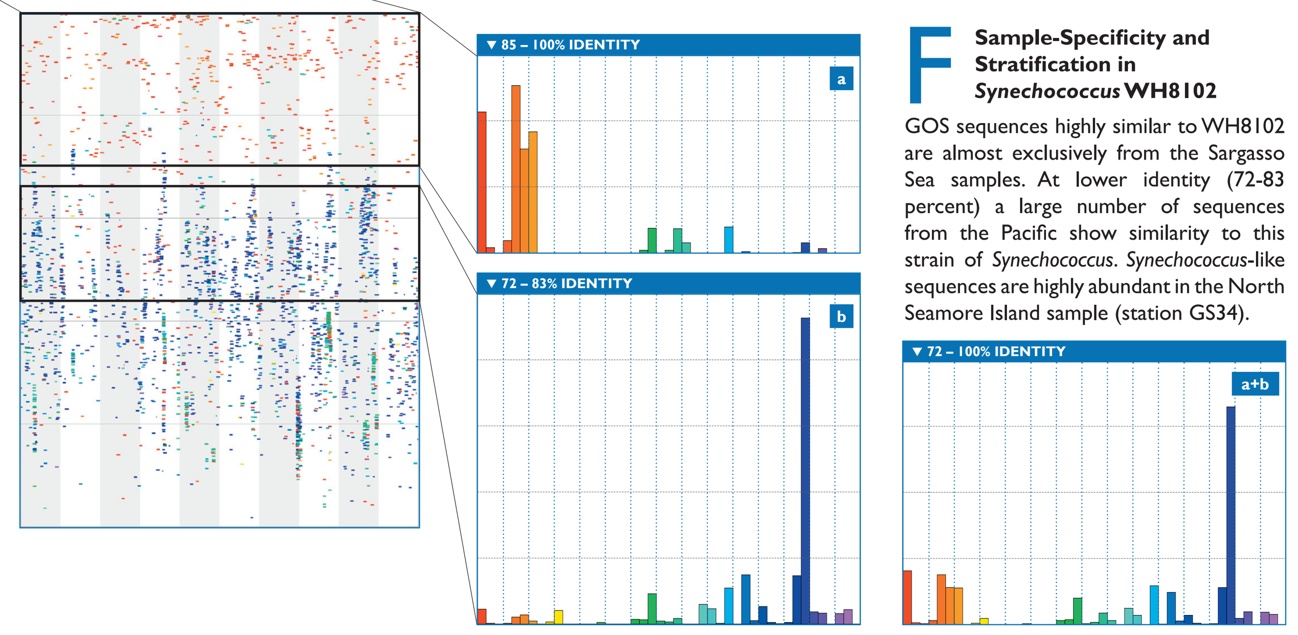

G Possible Recombination Event

Over most portions of the *P. marinus* MIT9312 genome, shredded MED4 sequences (black) are recruited in a pattern very similar to the GOS sequences even producing a zigzag, albeit at significantly lower identity. However, there are sections of MIT9312 where this is not true: MED4 can be equally similar or even more similar than the GOS sequences. The region shown here contains the two largest segments where this occurs. Phylogenetic analysis shows that the GOS, MED4, and MIT9312 sequences are equally divergent over this portion of the genome. Based on the tiling and mate pairing of both the MED4 and GOS sequences these segments are not associated with a break in synteny. The sudden shift in MED4 identity is plausibly the result of ancient recombination event or unknown functional pressures.

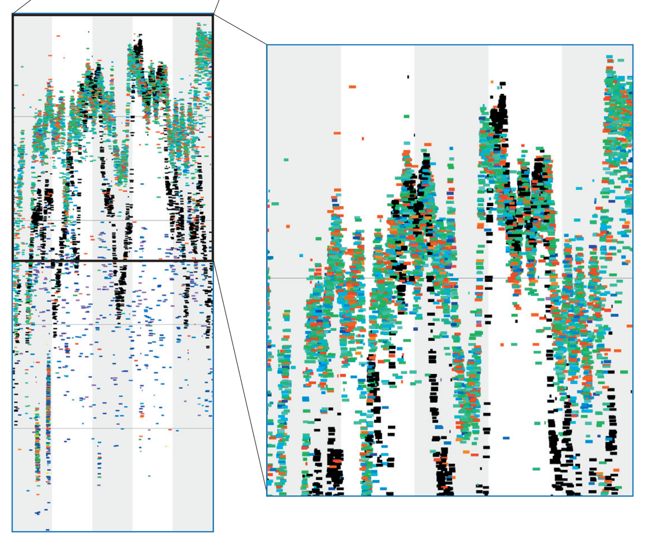

H Pacific Excluded Genomic Segment

This segment of the genome is abundant in the Atlantic, Gulf of Mexico, and Caribbean but greatly reduced from Pacific samples. The presence of this segment is presumably ancestral given its presence in the outgroup MED4. This segment encodes the phosphate binding protein (*PstS*) gene as well as several transporter and hypothetical proteins. A similar Pacific excluded segment is found on the HTCC1062 genome.

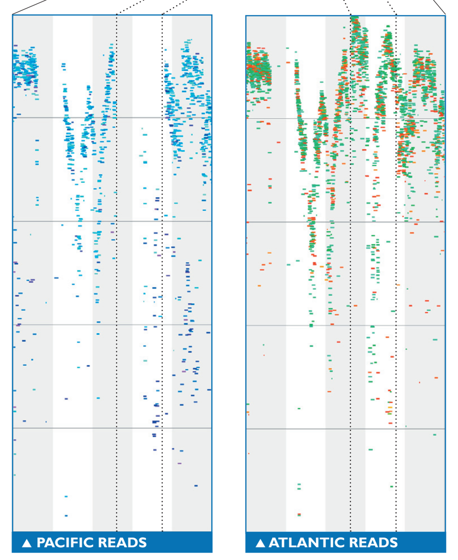

I Sample-Specificity and Stratification in *Prochlorococcus*

The majority of GOS sequences form a band that zigzags around 90 percent identity. The sequences that make up this band originate from a very consistent set of samples largely found in tropical equatorial waters and the Sargasso Sea. At lower identities (70-82 percent), sequences from the Pacific and to a lesser extent the Sargasso are much more abundant. These sequences are not prevalent enough to form a clear zigzag.

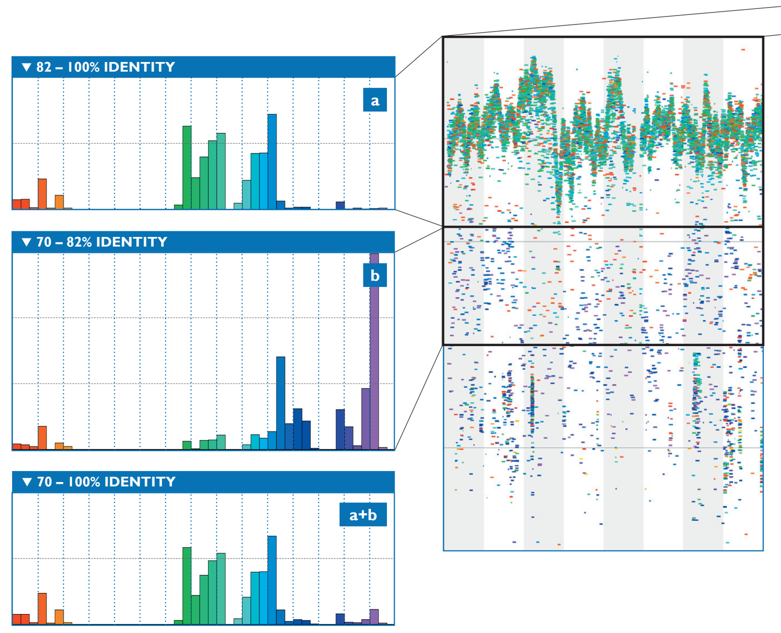

Mate Pairing Metadata Color Coding

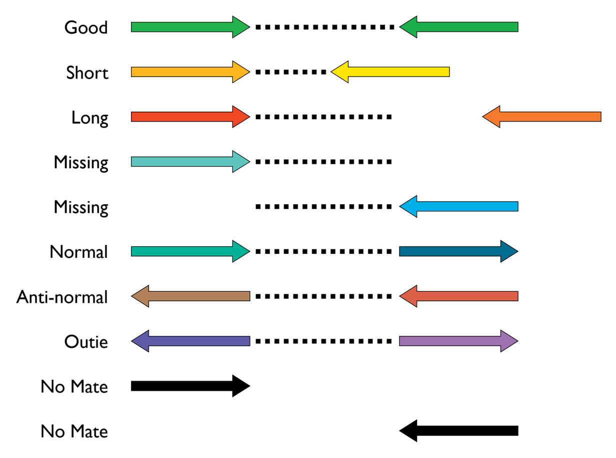

Sample Metadata Color Coding

00a) Sargasso Stations 13 and 11	04) Outside Halifax, Nova Scotia	13) Off Nags Head, NC	22) 250 miles from Panama City	32) Mangrove on Isabella Island
00b) Sargasso Stations 13 and 11	05) Bedford Basin, Nova Scotia	14) South of Charleston, SC	23) 30 miles from Cocos Island	33) Punta Cormorant, Hypersaline Lagoon, Floreana Island
00c) Sargasso Station 3	06) Bay of Fundy, Nova Scotia	15) Off Key West, FL	25) Dirty Rock, Cocos Island	34) North Seamore Island
00d) Sargasso Station 13	07) Northern Gulf of Maine	16) Gulf of Mexico	26) 134 miles NE of Galapagos	35) Wolf Island
01a) Hydrostation S	08) Newport Harbor, RI	17) Yucatan Channel	27) Devil's Crown, Floreana Island	36) Cabo Marshall, Isabella Island
01b) Hydrostation S	09) Block Island, NY	18) Rosario Bank	28) Coastal Floreana	37) Equatorial Pacific TAO Buoy
01c) Hydrostation S	10) Cape May, NJ	19) Northeast of Colón	29) North James Bay, Santigo Island	47) 201 miles from French Polynesia
02) Gulf of Maine	11) Delaware Bay, NJ	20) Lake Gatun	30) Warm seep, Roca Redonda	51) Rangirora Atoll
03) Browns Bank, Gulf of Maine	12) Chesapeake Bay, MD	21) Gulf of Panama	31) Upwelling, Fernandina Island	MED4) *Prochlorococcus marinus* MED4 Strain Shredded

doi:10.1371/journal.pbio.0050077.sd001

SOME RIGHTS RESERVED

PLoS Biology is the world's premier peer-reviewed, open-access journal published by the Public Library of Science.

OPEN ACCESS Freely available online
Download this poster and its corresponding research article at www.plosbiology.org

Credits: Douglas B. Rusch, Matthew LaPointe, Aaron L. Halpern, Karla Heidelberg, Marvin Frazier, Robert Strausberg, Robert Friedman, and J. Craig Venter
